# Human Retinal Organoids Provide a Suitable Tool for Toxicological Investigations: A Comprehensive Validation Using Drugs and Compounds Affecting the Retina

**DOI:** 10.1093/stcltm/szab010

**Published:** 2022-02-19

**Authors:** Birthe Dorgau, Maria Georgiou, Alexander Chaudhary, Marina Moya-Molina, Joseph Collin, Rachel Queen, Gerrit Hilgen, Tracey Davey, Philip Hewitt, Michael Schmitt, Stefan Kustermann, Francois Pognan, David H Steel, Evelyne Sernagor, Lyle Armstrong, Majlinda Lako

**Affiliations:** Newcastle University, Biosciences Institute, Faculty of Medical Sciences, Newcastle upon Tyne, UK; Newcells Biotech, Biosphere, Newcastle Helix, Newcastle upon Tyne, UK; Newcastle University, Biosciences Institute, Faculty of Medical Sciences, Newcastle upon Tyne, UK; Newcastle University, Biosciences Institute, Faculty of Medical Sciences, Newcastle upon Tyne, UK; Newcastle University, Biosciences Institute, Faculty of Medical Sciences, Newcastle upon Tyne, UK; Newcells Biotech, Biosphere, Newcastle Helix, Newcastle upon Tyne, UK; Newcastle University, Biosciences Institute, Faculty of Medical Sciences, Newcastle upon Tyne, UK; Newcastle University, Biosciences Institute, Faculty of Medical Sciences, Newcastle upon Tyne, UK; Newcastle University, Biosciences Institute, Faculty of Medical Sciences, Newcastle upon Tyne, UK; Northumbria University, Applied Sciences, Faculty of Health and Life Science, Newcastle upon Tyne, UK; Newcastle University, Biosciences Institute, Faculty of Medical Sciences, Newcastle upon Tyne, UK; Electron Microscopy Research Services, Newcastle University, Newcastle upon Tyne, UK; Merck Healthcare KGaA, Darmstadt, Germany; Merck Healthcare KGaA, Darmstadt, Germany; Pharmaceutical Sciences, F. Hoffmann-La Roche, Pharma Research and Early Development, Roche Innovation Center Basel, Switzerland; Novartis, PreClinical Safety, Basel, Switzerland; Newcastle University, Biosciences Institute, Faculty of Medical Sciences, Newcastle upon Tyne, UK; Newcastle University, Biosciences Institute, Faculty of Medical Sciences, Newcastle upon Tyne, UK; Newcastle University, Biosciences Institute, Faculty of Medical Sciences, Newcastle upon Tyne, UK; Newcells Biotech, Biosphere, Newcastle Helix, Newcastle upon Tyne, UK; Newcastle University, Biosciences Institute, Faculty of Medical Sciences, Newcastle upon Tyne, UK

**Keywords:** human embryonic stem cells, human induced pluripotent stem cells, retina, toxicology, retinal organoids, drug screening

## Abstract

Retinal drug toxicity screening is essential for the development of safe treatment strategies for a large number of diseases. To this end, retinal organoids derived from human pluripotent stem cells (hPSCs) provide a suitable screening platform due to their similarity to the human retina and the ease of generation in large-scale formats. In this study, two hPSC cell lines were differentiated to retinal organoids, which comprised all key retinal cell types in multiple nuclear and synaptic layers. Single-cell RNA-Seq of retinal organoids indicated the maintenance of retinal ganglion cells and development of bipolar cells: both cell types segregated into several subtypes. Ketorolac, digoxin, thioridazine, sildenafil, ethanol, and methanol were selected as key compounds to screen on retinal organoids because of their well-known retinal toxicity profile described in the literature. Exposure of the hPSC-derived retinal organoids to digoxin, thioridazine, and sildenafil resulted in photoreceptor cell death, while digoxin and thioridazine additionally affected all other cell types, including Müller glia cells. All drug treatments caused activation of astrocytes, indicated by dendrites sprouting into neuroepithelium. The ability to respond to light was preserved in organoids although the number of responsive retinal ganglion cells decreased after drug exposure. These data indicate similar drug effects in organoids to those reported in in vivo models and/or in humans, thus providing the first robust experimental evidence of their suitability for toxicological studies.

## Graphical Abstract

**Graphical Abstract:**
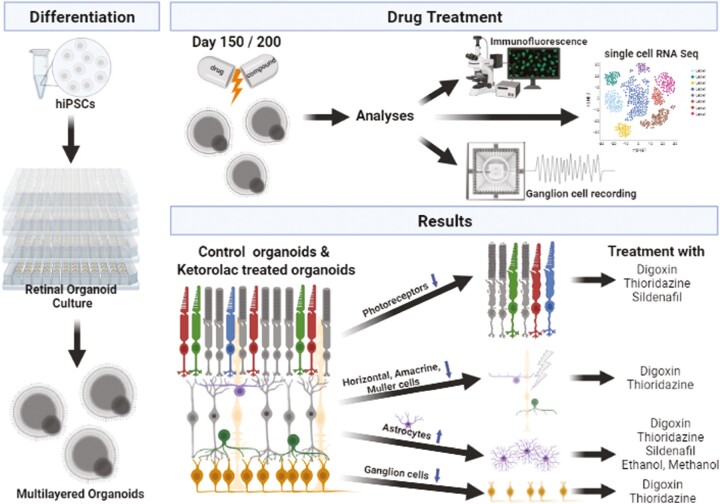

**Significance Statement**

Retinal drug toxicity testing is essential for the development of new treatments for various diseases. For this purpose, retinal organoids derived from human pluripotent stem cells provide an appropriate platform as they are akin to human retina. This study characterized the impact on retinal organoids of six well-known drugs (Digoxin, Thioridazine, Sildenafil, Ketorolac, Ethanol, and Methanol), demonstrating similar drug-related effects on organoids compared to those reported in vivo models and/or humans. This emphasizes the suitability of retinal organoids for in vitro toxicological studies and enables the development for new therapies in a wide range of diseases.

## Introduction

Assessing retinal drug toxicity has become important as different molecules and antibodies have become available for the treatment of neurodegenerative disorders.^1,2^ To date most drug and toxicology studies are performed in vivo in rodent models, but this approach is not optimal because there are anatomical and functional differences between rodent and human retina.^3^ For example, the rod-dominated mouse retina has only 2 types of cones (S- and M-cones) in contrast to the human retina, containing 3 different types of cones (S-, M-, and L-cones) densely packed in the fovea, an essential area for high-acuity color vision in humans and affected in key retinal diseases such as age-related macular degeneration. Light-responsive 3D retinal organoids derived from human pluripotent stem cells (hPSCs) provide an optimal platform for testing the efficacy and toxicity of drugs as they are similar to the human retina.^4-11^ They offer a closer model to the human retina than mice, eg, containing all retinal cell types including all 3 cone subtypes albeit not clearly arranged in a fovea. These 3D retinal organoids can be derived from patients with various age-related and inherited pathological conditions, enabling studies that focus on the correlation between diseases and response to various drugs. Importantly, such organoids provide the *in vitro* disease models that recapitulate diversity of the human disease, avoiding the generation of animal models with targeted mutations as in current practice.

To date, very few studies have validated the application of human retinal organoids for drug and toxicology screening. In 2018, moxifloxacin, a broad-spectrum antibiotic, was tested on retinal organoids, indicating that, similar to adult mouse retina, the primary affected cell types were photoreceptors.^6^ Additionally, Saengwimol and colleagues^12^ used a retinoblastoma retinal organoid model to assess the effect of well-known chemotherapy drugs, and recently, Liu et al.^13^ also tested the effect of spleen tyrosinase kinase (SYK) inhibitors on a retinoblastoma organoid model, displaying a significant therapeutic response. Notwithstanding, a thorough validation of hPSC-derived retinal organoids for drug and toxicology screening has not been performed to date and forms the thrust of the work described herein. We used two different human-induced pluripotent stem cell (hiPSC) lines to test the effects of known compounds, namely digoxin, thioridazine, sildenafil, ethanol, and methanol, all of which have been associated with ocular related side effects (Table 1). In addition, we included a control group by exposing retinal organoids to ketorolac, a nonsteroidal anti-inflammatory drug (NSAID), which is used as a short-term treatment for moderate to severe pain,^14^ and shown to have no effect on the retina.^14,15^

**Table 1.** Drugs, their applications, ocular symptoms, and impact on retinal cells.

Drug	Type of compound	Clinical indications for use/known systemic effects of exposure	Administration/ used concentration	Ocular symptoms/effects	Impact on retinal cells
**Ketorolac**	Non-steroid anti-inflammatory drug	**•** Antipyretic and analgesic activity **• **Anti-inflammatory for chronic rheumatoid arthritis, osteoarthritis, ankylosing spondylitis, and a wide range of other inflammatory conditions	**Human** **• **Intravenous: 30 mg, every 6 h as needed (maximum dose: 120 mg/day) **Rabbit** **• **Intravitreal: 125mg-1 mg (single dose)	**• **No reported ocular symptoms	
**Digoxin**	Cardiac glycoside	Cardiac arrhythmias include atrial fibrillation, atrial flutter, and as a reserve drug for cardiac failure	**Human** **• **Oral dosage: 0.125-0.25 mg/day **• **Serum level therapeutic range (0.5-2ng/mL) **Monkey** **• **Intravenous: 0.1 mg/kg **Mouse** **• **Intraperitoneal: 1 or 2 mg/kg (14 days) **• **Intraperitoneal: 0.1—2 mg/kg (3 days)	**• **Blurred vision **• **Central scotomas or seeing yellowish, bluish, greenish **• **Decreased **• **Visual acuity and color vision **• **Visual hallucinations **• **Dyschromatopsia	**Monkey** **• **Cone system (changes in photopic ERG) **Mouse** **• **Photoreceptors **• **Retinal degeneration **• **Photoreceptor induced activation of Müller cells
**Thioridazine**	Antipsychotic drug	Psychoses, including schizophrenia	**Human** **• **Oral: 800 mg/day or below	**• **Blurred vision **• **Reddish or brownish discoloration of vision **• **Nyctalopia **• **Optic atrophy **• **Decrease in visual acuity **• **Visual fields deficits **• **Pigmentary granularity	**Human** **• **Reduced photopic & scotopic a-/b-waves **• **Sometimes decreased oscillatory potentials **• **Disorganization and loss of photoreceptor outer segments followed by loss of RPE **• **macrophages present
**Sildenafil**	Phosphodiesterase-5 inhibitor	Erectile dysfunctions and pulmonary arterial hypertension	**Zebrafish** **• **1-100 µM (3 days) **Rat** **• **Oral: 10 mg/kg/day (8 weeks) **Mouse** **• **Intraperitoneal: 7.25-72.5 mg/kg (single dose) **Monkey** **• **Intravenous: 1-3 mg/kg **Human** **• **Oral: 25-100 mg/day (short term)	**• **Multicolored photopsia **• **Erythropsia (red-tinted vision) **• **Bluish vision **• **Increased perception of brightness **• **Blurred vision **• **Significant dilatation of retinal blood vessels **• **Ocular pain, headache *Less common:* **• **Nonarteritic ischemic optic neuropathy **• **Reversible increase in intraocular pressure	**Zebrafish** **• **Reduce rod outer segment shedding **Rat** **• **Vacuolations of the photoreceptor outer segments **• **Morphological change of **Müller cells** **• **Decreased cellular populations particularly in the INL & GCL **Mouse** **• **Decreased ERG responses **Monkey** **• **a- and b-wave changes in ERG **Human** **• **ERG changes, more pronounced in the cone pathway than in the rod pathway (decreases a/b-waves, prolonged implicit times)
**Ethanol**	Alcohol	Fetal alcohol exposure (FAE)	**Rat** **• **Subcutaneous: 2.5 g/kg (2 doses) **Monkey** **• **Intravenous: 2.15 g/kg followed by 0.2 g/kg every hour for 6 h **• **Drinking water: 1.2-5.51 g/kg (daily from E95 until birth) **Mouse** **• **Intraperitoneal: 3.48 g/kg at E8 (2 doses) **• **Intragastric gavage: 2 or 4 g/kg/day (E5 to parturition) **Zebrafish** **• **Water: 0.25-2% EtOH/day either from gastrulation until hatching or from gastrulation until postnatal day 6 **• **Water: 100 mM or 150 mM **• **Water: 262.5 mM for 24, 48, and 72 h post-fertilization **Chick** **• **Injection of embryo: 0.5 mg at E6 (single dose) **Human** **• **No alcohol is recommended during pregnancy	**• **Microphthalmia and photoreceptor differentiation defects (zebrafish) **• **Astrogliosis in GCL (monkey) **• **Optic nerve hypoplasia (Mouse)	**Rat:** **• **Ganglion cells **Monkey** **• **Strong astrogliosis in GCL **• **Increased photosensitivity of cone photoreceptors **Mouse** **• **Retinal progenitor cells stay in a premature cell-cycle arrest **• **Bbipolar and horizontal cells **• **Disruption of OLM (MC end-feet) **Zebrafish:** **• **Photoreceptors **• **Müller cells **Chick:** **• **Ganglion cells **• **Müller cells **• **RPE **• **Cells in IPL **• **Optic nerve
**Methanol**	Alcohol	Initial transient central nervous system depression, followed by an asymptomatic latent period lasting 12 to 24 hours	**Human** **• **Oral: a lethal dose of pure methanol: 30-240 mL or 1 g per kg **• **Respiratory intake: a lethal dose of pure methanol: 4000-13,000 mg/L **Monkey:** **• **Intramuscular: 5-6 g/kg (single dose) **• **Intramuscular: 2 mg/kg (multiple doses) **Rat** **• **Intraperitoneal: 4 k/kg followed by 2 doses of 2 g/kg after 24 h and 48 h respectively **• **Intragastric gavage: 8 mL/kg followed by a dose of 2 mg/kg after 24 h	Development of formic acidaemia, uncompensated metabolic acidosis, visual toxicity, coma, and sometimes, death	**Human** **• **Optic nerve swelling **• **Vision defect to blindness **• **Ganglion cell complex (GCC) and RNFL became thinner **• **Oscillatory potentials (ERG) decreased **• **Focal RPE detachments **• **An enlarged subretinal space **• **Swollen apices of RPE cells **Monkey:** Optic disc edema **Rat** **• **Photoreceptors **• **Attenuated ERG responses

The cardiac glycoside, digoxin, is used to treat atrial fibrillation or flutter and it acts as an inhibitor of Na^+^/K^+^ ATPase pump in the plasma membrane, resulting in an increased intracellular Na^+^ and Ca^2+^, which subsequently promotes cardiac muscle contraction.^16^ Ocular side-effects reported by patients include blurred vision, visual disturbances including central scotomas and a yellowish tinge to the vision and visual hallucinations (Table 1). It is thought that digoxin primarily affects photoreceptors since digoxin intoxication leads to prolonged implicit times and abnormal amplitudes in the cone-mediated electroretinogram (ERG) and irregularities in color vision.^17-21^ Thioridazine, a piperidine typical antipsychotic drug, was used to treat psychoses, mainly schizophrenia in the past.^22^ Ocular manifestations (Table 1) including changes in color vision and granular pigmentary retinopathy are thought to be due to either binding of Thioridazine to melanin granules in RPE, inhibition of key retinal enzymes causing oxidative phosphorylation abnormalities in rhodopsin synthesis, and/or blocking of dopamine receptors leading to an increase in melatonin synthesis.^23-26^ A common drug usually administered to treat erectile dysfunction is sildenafil (also known as Viagra), a phosphodiesterase (PDE) 5 inhibitor (Table 1). Sildenafil-induced retinal side effects have been reported in human^27-30^ and animal studies,^31-34^ including changes of color vision and light perception, photophobia, and visual disturbances (Table 1).

Ethanol and methanol, both belonging to the group of alcohols, affect the retina in different ways. While ethanol is predominantly deleterious during retinal development, methanol can affect the retina via conversion to formic acid, causing damage at any time during development and adulthood (Table 1). Fetal alcohol exposure (FAT) caused by ethanol can lead to various ocular side effects including ERG abnormalities, microphthalmia, and optic nerve hypoplasia in humans.^35,36^ In animal models photoreceptor differentiation defects, astrogliosis in ganglion-cell layer (GCL), and adverse effects in RPE, and inner nuclear layer (INL) have been reported after ethanol exposure^37-43^ (Table 1). Methanol can accumulate in the vitreous body, and depending on the severity of intoxication, ocular symptoms can vary from mild/moderate effects including vision defects, ERG abnormalities, and optic disc edema, and up to acute irreversible blindness^44-49^ (Table 1).

In this manuscript, we have fully characterized retinal organoids from two different hiPSC lines using a combination of quantitative immunofluorescence (IF), scanning electron microscopy, neural activity recordings from retinal ganglion cells (RGCs), and single-cell RNA-Seq. Our data demonstrate that hiPSC retinal organoids contain all retinal cell types organized in multiple nuclear and synaptic layers, with well-aligned and mature photoreceptors clearly visible in the putative outer nuclear layer (ONL). Application of digoxin, thioridazine, and sildenafil led to a significant increase in cell death in horizontal, amacrine, and ganglion cells. Müller cell organization was impaired by digoxin and thioridazine. Retinal organoids showed light-induced activity changes of RGCs even after drug exposure, although the number of responding RGCs was reduced after drug treatments. Importantly, astrocyte proliferation and sprouting in response to digoxin, thioridazine, and sildenafil exposure, suggests that these drugs induce severe gliosis. In conclusion, we demonstrate that hiPSC-derived retinal organoids display comparable effects to in vivo published data for these retinotoxic compounds, validating their use for large-scale drug and toxicology screens.

## Materials and Methods

### Human Pluripotent Stem Cell Culture and Retinal Organoid Differentiation

**Figure 1.**
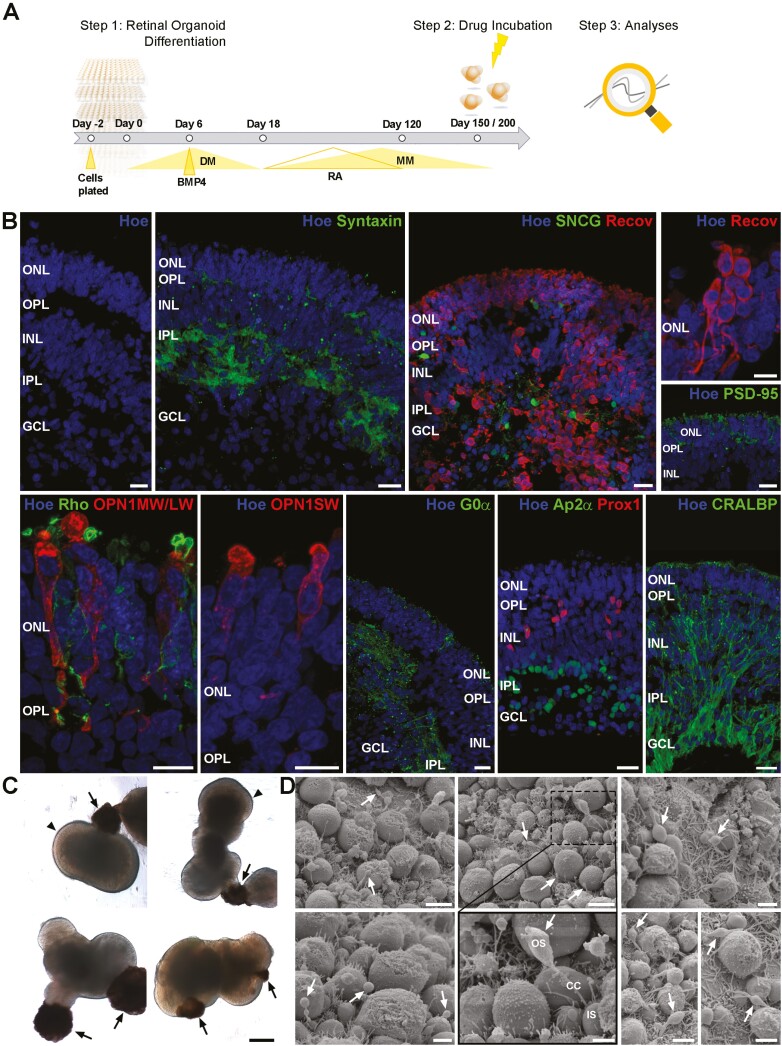
Experimental design and characterization of multilayered retinal organoids used in the study. (**A)**: Schematic timeline of the experimental procedure. (**B)**: Nuclear staining (Hoe; blue) and syntaxin immunofluorescence (green) showed layering of retinal organoids, containing ONL, OPL, INL, IPL, and GCL at day 150 of differentiation. Densely packed photoreceptors (Recov; red) are in the ONL, showing all subcellular compartments illustrated by Recov (red) and PSD-95 immunolabeling (green). Retinal organoids comprise all photoreceptor subtypes, Rods (Rho; green), middle-/long-wavelength cones (OPN1MW/LW; red), and short-wavelength cones (OPN1SW; red). The INL contains bipolar cells (G0α; green), horizontal cells (Prox1; red), amacrine cells (Ap2α; green), and Müller cells (CRALBP; green). Retinal ganglion cells (SNCG; green) were found in the basal side of retinal organoids. Nuclei were counterstained with Hoechst (blue). (**C)**: Representative brightfield images of retinal organoids derived from WT3 indicated RPE patches (arrows) next to the neuroepithelium and developing photoreceptor IS/OS (arrowheads) at the apical edge of organoids. (**D)**: Scanning electron microscopy illustrated photoreceptor IS, connecting cilia (CC) and developing OS at day 200 of differentiation. Abbreviations: GCL, ganglion-cell layer; Hoe, Hoechst; INL, inner nuclear layer; IPL, inner plexiform layer; IS; inner segment; ONL, outer nuclear layer; OPL, outer plexiform layer; OS, outer segment; Recov, Recoverin; Rho, Rhodopsin. Scale bars, 20 μm for B and C; 5 μm or 2 μm for D.

Two hiPSC lines named WT3^6^ and WT4 derived from skin fibroblasts of healthy males (reprogramming method: Sendai virus) were expanded in mTESR 1 (StemCell Technologies, 05850) on growth factor reduced Matrigel (BD Biosciences, San Jose, CA) coated plates at 37 °C and 5% CO_2_. Retinal organoids were generated as described by Georgiou et al^50^ with some modifications (Fig. 1A): The media was supplemented with 1.5 nM BMP4 (R&D, 314-BP) on day 6 and with 10% fetal calf serum (FCS; Life Technologies, UK) and retinoic acid (RA; 0.5 µM; Sigma-Aldrich) after day 18 of differentiation. RA was added until day 120 of differentiation and retinal organoids were further cultured until day 150 and/or 200 of differentiation. The media was changed every 2-3 days.

### Toxicological Experiments

The number of independent biological experiments for toxicological experiments is *N* = 3, including WT3-derived retinal organoids (*N* = 2), and WT4-derived organoids (*N* = 1). WT3-derived retinal organoids were used as the main cell line for all experiments, which were further validated in WT4-derived organoids apart from ethanol exposure. All experiments were performed on day 150 of differentiation, with exception of single-cell (sc) RNA-Seq and scanning electron microscopy (SEM) which were performed on day 200 (Fig. 1A). The following drugs were tested in this study: ketorolac, digoxin, thioridazine, sildenafil, ethanol, and methanol (please refer to Table 2 for tested concentrations). Stock solutions for each drug were prepared in phosphate-buffered saline (PBS) or dimethyl sulfoxide (DMSO) with the latter being further diluted in PBS before use. Alongside all drugs, an additional control ([PBS] supplementation) was used in all experiments.

**Table 2.** Drugs, their concentrations, and exposure time used for toxicological experiments.

Drug	Concentration	Exposure time	Company
Ketorolac	2.5 mM	24 h	Sigma-Aldrich, K1136
Digoxin	40 nM	24 h	Sigma-Aldrich, D6003
Thioridazine	135 µM	24 h	Sigma-Aldrich, T9025
Sildenafil	225 µM	7 days	Sigma-Aldrich, PZ0003
Ethanol	500 mM	24 h	VWR, 20821.33
Methanol	32 mM	24 h	VWR, 20846.326

On day 149 or 199 of differentiation retinal organoids were exposed to the test drugs for 24 h, except sildenafil (Table 2). Sildenafil incubation was carried out over 7 days (start day of exposure: day 143 or 193), supplementing the medium with fresh sildenafil at each media change. Brightfield images of retinal organoids were taken before and after drug exposure using a Zeiss AxioVert1 (Zeiss, Germany) with a 5×/0.15 air objective. After drug exposure, retinal organoids were fixed and embedded for IF analysis or used fresh for electrophysiological recordings from the RGC layer or scRNA-Seq. The supernatant was collected for a lactate dehydrogenase (LDH) Cytotoxicity Assay.

### Quantification of Neuroepithelium Thickness

For each condition, brightfield images (*n* = 7) were taken before and after drug exposure of WT3-derived retinal organoids on day 150 of differentiation. Measurement of the neuroepithelium thickness was performed using FIJI,^51^ including 8 individual measurements of the neuroepithelium thickness per organoid image. Excel and Prism (GraphPad, USA) were used for further analysis. The mean and standard errors of all means (SEM) were calculated for all conditions and statistical significance was tested using one-way ANOVA (Bonferroni statistical hypothesis for multiple test correction). Statistical differences were defined as followed: ∗*P* < .05, ∗∗*P* < .01, ∗∗∗*P* < .001, ∗∗∗∗*P* < .0001.

### LDH Cytotoxicity Assay

To determine the cytotoxic effect of each drug (Table 2) on retinal organoids, the Pierce LDH Cytotoxicity Assay Kit (Fisher Scientific, 88954) was used according to the manufacturer’s manual. All conditions were set out as triplicates. The absorbance was measured at 490 nm and 680 nm using a Varioskan LUX plate reader (Thermo Fisher Scientific, UK). Excel and Prism (GraphPad, USA) were used for further statistical analysis. The mean and standard errors of all means (SEM) were calculated for all conditions.

### Immunofluorescence

Retinal organoids (10-15) from control and all drug conditions were collected after drug exposure on day 150 of differentiation and further processed for IF by fixation in 4% (w/v) paraformaldehyde (PFA) for 20-30 minutes followed by several washing steps in PBS. After cryoprotection in PBS containing 30% sucrose overnight, organoids were embedded in OCT (Cell Path Ltd., Newtown, UK), sectioned (10 µm) on a cryostat (Leica Cm1860), and stored at −20°C until further use.

After air-drying, sections were rinsed in PBS and incubated for 1 h at room temperature in blocking solution (10% normal goat serum, 0.3% Triton–X-100 in PBS). All antibodies were diluted in antibody diluent solution [ADS; 1% bovine serum albumin (BSA), 0.3% Triton–X-100 in PBS]. Primary antibodies (Table 3) were applied overnight at 4°C. After several washing steps in PBS, sections were incubated with secondary goat antibodies conjugated either to Alexa488 (Jackson Immuno Research Laboratories) or Cy3 (Jackson Immuno Research Laboratories) for 2 h at room temperature in the dark. Sections were then washed several times in PBS and mounted with VectaShield (Vector Laboratories, Burlingame, CA) containing Hoechst (Life Technologies). For each antibody a control IF was carried out by omitting the primary antibody.

**Table 3.** List of primary antibodies used for immunofluorescence.

Antibody	Host and clonality	Dilution	Company, Cat. No
AP2α, clone 3B5	Mouse, monoclonal	1:100	Santa Cruz Biotechnology Inc., sc-12726
Casp-3	Rabbit, polyclonal	1:400	Cell Signaling, 9661
CRX, clone 4G11	Mouse, monoclonal	1:200	Abnova, H00001406-M02
CRALBP, clone B2	Mouse, monoclonal	1:100	Abcam, ab15051
Α-crystallin B (CRYAB)	Rabbit, polyclonal	1:500	Abcam, ab76467
G0α, clone 2A	Mouse, monoclonal	1:500	Millipore, MAB3073
Gαt1, K-20	Rabbit, polyclonal	1:200	Santa Cruz Biotechnology Inc., sc-389
GFAP	Rabbit, polyclonal	1:1000	Dako, Z0334
HuC/HuD, clone 16A11	Mouse, monoclonal	1:200	Invitrogen, A21271
Opsin red/green	Rabbit, polyclonal	1:200	Millipore, ab5405
Opsin blue	Rabbit, polyclonal	1:200	Millipore, ab5407
PSD-95	Mouse, monoclonal	1:500	Millipore, MAB1598
Prox1	Rabbit, polyclonal	1:1500	Millipore, ab5475
Recoverin	Rabbit, polyclonal	1:1000	Millipore, ab5585
Rhodopsin, clone 1D4	Mouse, monoclonal	1:200	Millipore, MAB5356
SMI-32	Mouse, monoclonal	1:200	Covance, SMI-32P
SNCG	Mouse, Monoclonal	1:500	Abnova, H00006623-M01A
Syntaxin, clone HPC-1	Mouse, monoclonal	1:200	Sigma-Aldrich, S0664

### Image Acquisition

Images were taken using a Zeiss Axio ImagerZ2 equipped with an Apotome.2 and Zen 2012 blue software (Zeiss, Germany). Scanning of image stacks was performed with either a 20×/0.8 air objective or a 63×/1.4 oil immersion objective using a *z*-axis increment of either 0.49 µm for 20× air objective or 0.24 µm for 63× oil immersion objective. Different retinal organoids (5-10) from each independent biological replicate (*N* = 3) were imaged for all conditions. Final images are displayed as a maximum projection and adjusted for brightness and contrast in Adobe Photoshop CS6 (Adobe Systems).

### IF Image Quantification

IF image quantitation with 5-10 examples per condition was performed as described by Dorgau et al. ^5^ using the MATLAB software (Mathworks, MA, USA). Data were plotted and statistically analyzed using Prism (GraphPad, USA). The mean (±SEM) was calculated for all conditions and statistical significance was tested using one-way ANOVA (Bonferroni statistical hypothesis for multiple test correction). ∗*P* <.05, ∗∗*P* <.01, ∗∗∗*P* <.001, ∗∗∗∗*P* <.0001. Statistical significance tests were performed for all drug conditions (ketorolac, digoxin, thioridazine, sildenafil, ethanol, and methanol) against the control-treated group and results are shown in graphs. Additionally, statistical significance differences were also carried out using the ketorolac-treated group as a reference against all other drug conditions (digoxin, thioridazine, sildenafil, ethanol, and methanol). Results from both tests are presented in Supplementary Table S1.

### RGC Recordings

RGC recordings were carried out immediately after stopping drug exposure experiments. Retinal organoids (6-8) derived from WT3 and WT4 per condition were used. Electrophysiological recordings were performed as described.^5^ Briefly, retinal organoids were incubated with 9-*cis* retinal (10 nM; Sigma-Aldrich, UK) overnight before recordings. After several washes in artificial cerebrospinal fluid (aCSF) (in mM: 118 NaCl, 25 NaHCO_3_, 1 NaH_2_PO_4_, 3 KCl, 1 MgCl_2_, 2 CaCl_2_, 10 glucose, 0.5 L glutamine, and 0.01 9-*cis*-retinal) organoids were opened longitudinally and placed facing down with the presumed RGC layer onto a 4096 channel multielectrode array (MEA). The organoids settled down for 2-3 h before recordings, using the BioCam4096 MEA platform with HD-MEA Arena chips (3Brain GmbH, Lanquart, Switzerland). After recording spontaneous activity in the dark for 5 min full-field white light pulses (WLP, 200 ms, 217 µW/cm^2^ irradiance, 1 Hz) were flashed for 5 min onto the retinal organoids.

Extraction of spikes from raw traces was done using a quantile-based event detection^52^ and single-unit spikes were sorted by an automated spike sorting method for dense, large-scale recordings.^53^ Firing rate analyses were evaluated using MATLAB (Mathworks, MA). RGCs were considered responsive if they changed their spiking activity for at least 25% (increase or decrease) during 30 s after WLP onset compared to the similar time window before the light stimulus (dark condition). The mean percentage change (±SEM) in activity between windows was calculated and the percentage of responsive cells after WPL was validated relative to the control condition (100%). Plots and statistical significance tests (Mann-Whitney test) were performed using Prism (GraphPad, CA).

### scRNA-Seq analysis

Retinal organoids were dissociated to single cells using the Neurosphere Dissociation kit (P) (Miltenyi Biotech) according to the manufacturer’s protocol. Cell capture and library generation were carried out using the Chromium Single Cell 3ʹ Library & Gel Bead Kit, version 3 (10× Genomics). scRNA-Seq libraries were sequenced to 50,000 reads per cell on an Illumina NovaSeq 6000. CellRanger version 3.01 was used to de-multiplex, align the reads to human reference genome GRCh38 and create a gene expression matrix. Quality control checks were performed and any cells where fewer than 1000 reads or 500 genes or greater than 15% mitochondrial reads were removed from the analysis. DoubletFinder (version 2.0.3) was used to find and remove doublets.

Each sample was log normalized using Seurat (version 3.1.3). The top 2000 highly variable genes and PCA dimension reduction were applied to the data. The first 30 principal components were used to integrate the datasets using the standard Seurat integration approach which corrects for batch effects. The combined datasets were then clustered using a resolution of 0.5. Marker genes for each cluster were identified using the Wilcoxon test within Seurat. Clusters were assigned with cell type using these marker genes. Uniform manifold approximation and projection (UMAP) plots were used to visualize the clusters. Selected cell types were extracted from the full dataset and then re-clustered at a resolution of 0.5 using the method above. Single-cell RNA-Seq data were deposited to GEO (GSE172138).

Differential expression analysis was performed to look for changes in gene expression between control organoids (PBS treated) and drug-treated organoids. A core analysis from Qiagen Ingenuity Pathway Analysis (IPA) was used to study disease function, canonical pathways associated with these differentially expressed gene lists. Cluster expression percentage plots were generated using Prism (GraphPad, CA), showing the mean for each condition. The black line represents the average of control conditions (PBS control and ketorolac) and the dashed black lines characterize their corresponding standard deviation (SD).

### Scanning Electron Microscopy (SEM)

WT3-derived retinal organoids were fixed in 2% glutaraldehyde (TAAB Laboratories Equipment Ltd, Aldermaston, UK) in Sorenson’s Phosphate Buffer for at least 24 h. After several washing steps in Sorenson’s phosphate buffer, an ethanol dehydration process was performed, starting with 25%, 50%, and 75% ethanol for 30 min followed by twice 100% ethanol incubations for 1 h each. The final dehydration was carried out with carbon dioxide in a Baltec Critical Point Dryer (Leica Geosystems Ltd, Milton Keynes, UK). Retinal organoids were then mounted on an aluminum stub with Achesons Silver Dag (Agar Scientific, Stansted, UK) and dried overnight. Then organoids were coated with gold, 5-10 nm, using a Polaron SEM Coating Unit (Quorum Technologies Ltd, Laughton, UK) and imaged using a Tescan Vega LMU Scanning Electron Microscope with a Tescan supplied software (Tescan, Girton, UK).

## Results

### Characterization of Retinal Organoids

**Figure 2.**
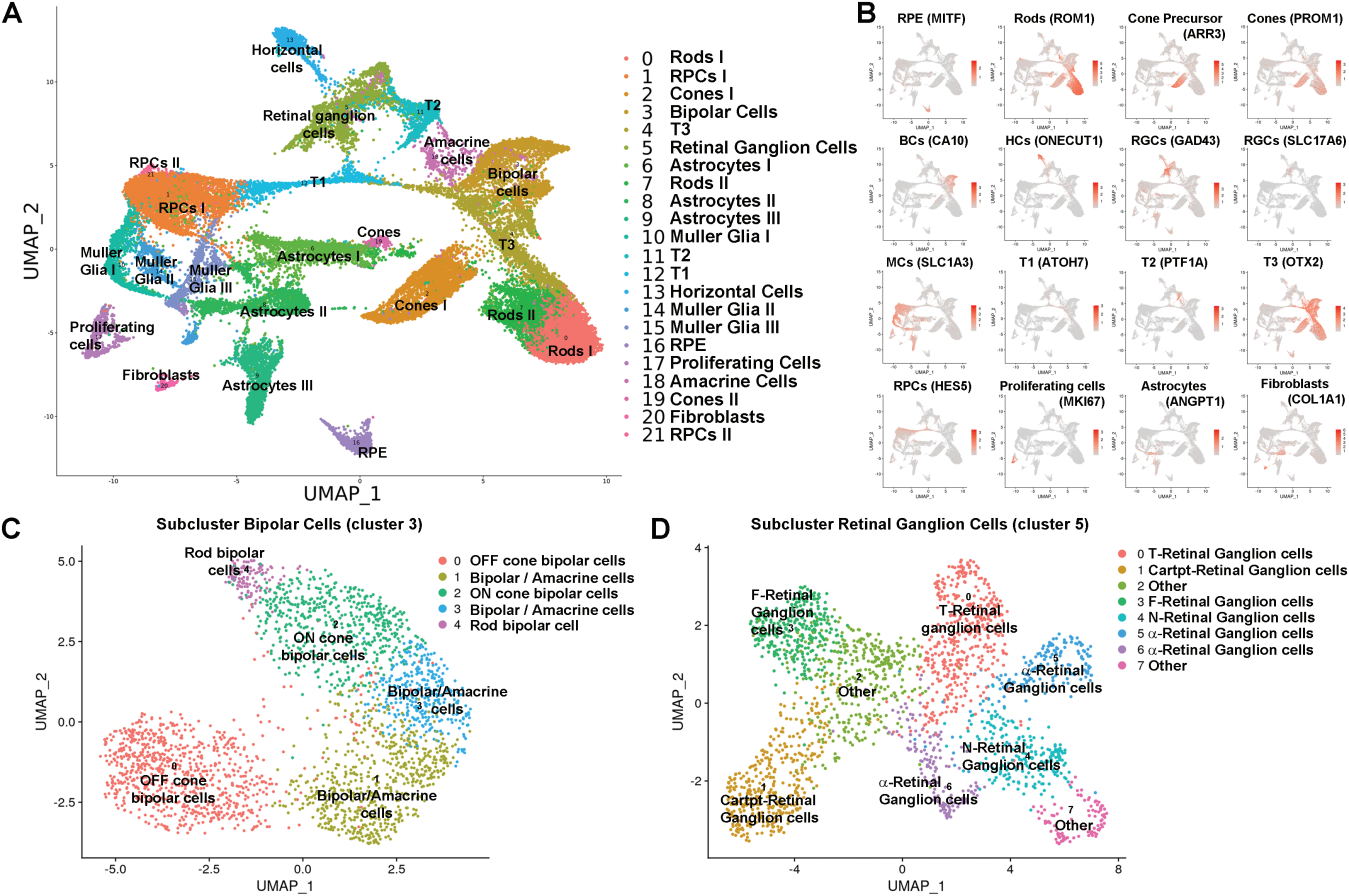
Single-cell RNA-Seq analyses of retinal organoids derived from WT3-iPSCs display all retinal cell types at day 200 of differentiation. All drug conditions and controls were included. (**A)**: UMAP maps showed 21 transcriptionally distinct cell clusters, comprising all the main retinal cell types together with non-retinal cell clusters, including a proliferating cell and a Fibroblast cell cluster. (**B)**: Example profiles of key retinal cell-type marker gene expression for individual clusters, enabling cluster definition in A. (**C)**: UMAP map of bipolar cell sub-cluster (3) revealed main bipolar cell subtypes: OFF Cone BC, ON Cone BC and Rod BC. (**D)**: UMAP map of retinal ganglion-cell sub-cluster (5) showed 7 different ganglion-cell subtypes.

Human retinal organoids were generated from two different hiPSCs (WT3 and WT4) and used either at day 150 or day 200 of differentiation (Fig. 1A). To validate their application for retinal toxicology studies, we performed an in-depth characterization by IF (Fig. 1B) at day 150 and SEM (Fig. 1D) and scRNA-Seq at day 200 of differentiation respectively (Fig. 2). At each time point, retinal organoids displayed 3 nuclear layers (ONL; inner nuclear layer, INL; ganglion-cell layer, GCL) separated by 2 synaptic layers (outer plexiform layer, OPL; inner plexiform layer, IPL) indicated by Syntaxin IF (Fig. 1B). The ONL at the apical edge of retinal organoids was densely packed with photoreceptors, labeled with Recoverin and/or PSD-95 (Fig. 1B). Photoreceptor subtypes, rods and cones, were observed in all retinal organoids, as indicated by rhodopsin expression for rods, middle/long-wavelength cone opsins (OPN1MW/LW) for green/red cones and short-wavelength cone opsins (OPN1SW) for blue cones. Photoreceptors subcellular compartments such as axons, synaptic terminals (Fig. 1B), connecting cilium, and inner and outer segments (IS/OS) were visible by light microscopy (Fig. 1C, arrowheads for OS) and SEM (Fig. 1D, arrows for OS). Bipolar cells (BCs) identified by G0α, horizontal cells (HCs) marked by Prox1 immunostaining and Ap2α-positive amacrine cells (ACs) were found in the INL (Fig. 1B). Within the INL, horizontal cells were more distal, close to the OPL, than amacrine cells. The latter was found more proximal, next to the IPL as well as in the GCL (displaced ACs). CRALBP-positive Müller glia cells (MCs) with their cell bodies in the INL stretched radially through retinal organoids, forming most likely the outer and inner limiting membrane (Fig. 1B). RGCs, immunostained for SNCG, were observed in the center of organoids, representing the GCL (Fig. 1B). Most retinal organoids (70-75%) developed RPE patches next to the neuroepithelium by day 150 of differentiation (Fig. 1C, arrows).

To assess the cellular composition of retinal organoids and gain additional insight into cell type and subtypes, scRNA-Seq was performed at a later stage of differentiation (day 200) (Fig. 2). After quality control and filtering, scRNA-Seq transcriptomes from 34,941 cells from WT3-derived retinal organoids (including all drug conditions) were obtained and merged using the Seurat package to allow the analysis of a higher cell number. Groups of transcriptomically similar cells were clustered and visualized using UMAP, revealing 21 distinct clusters (Fig. 2A). The percentage of cells in each cluster for each condition is listed in Supplementary Table S2. The highly and differentially expressed marker genes (Supplementary Table S3) were used to identify each cluster. The expression of key retinal cell–type-specific markers is shown in Fig. 2B. These 21 clusters comprised all main retinal cell types such as rods, cones, HCs, BCs, ACs, RGCs, MCs, and RPE alongside 2 clusters of retinal progenitor cells (RPCs) and transient progenitor cell clusters (T1, T2, and T3) (Fig. 2A). These transient progenitor cells were recently reported by Sridhar et al^54^ providing evidence that they give rise to RGCs (T1), horizontal and amacrine cells (T2), and bipolar (BCs) and photoreceptor cells (T3). Retinal organoids also contained non-neuronal cell clusters such astrocytes, fibroblasts, and a cluster of proliferating cells characterized by high expression of Ki67. Subclustering analyses were performed to assess the presence of cell subtypes within the bipolar and RGC cluster. Five different subclusters were found in bipolar cells (cluster 3) showing the presence of the main BC subclasses, namely the Rod ON-BCs, Cone ON-BCs, and Cone OFF-BCs besides 2 mixed clusters of bipolar and amacrine cells (Fig. 2C, Supplementary Table S4). Importantly, RGCs were present throughout the differentiation of retinal organoids (Figs. 1, 2, and 5). The subclustering of RGCs (cluster 5) following the cell type definition reported by Tran et al.^55^ revealed 8 RGC subclasses (Fig. 2D, Supplementary Table S5) as follows: 2 αRGCs (clusters 5 and 6), T-RGCs (cluster 0), Cartpt-RGCs (cluster 1), F-RGCs (cluster 3), N-RGCs (cluster 4), and 2 undefined clusters (‘Other’, clusters 2 and 7). In summary, these data show that hiPSC-derived retinal organoids generated herein resemble the structural morphology and cell-type composition of the human retina at day 150/200 of differentiation and thus are suitable as an *in vitro* model for toxicological experiments.

### Toxicological Experiments

This study used 5 compounds, namely digoxin, thioridazine, sildenafil, ethanol, and methanol, all of which have known retinal side-effects alongside a control drug namely ketorolac, where no vision-related side-effects are reported so far (please refer to Table 1 for side-effects). An additional control group (PBS-treatment) was included.

#### Assessment of retinal organoid specific drug concentrations

As each drug is used to treat different diseases in humans (please refer to Table 1 for application of drugs), their dose application is unique. We were guided by published studies of local drug administration in animal models (Table 1) to select a dose range for testing in the retinal organoids. Therefore, as a first step, we determined the optimal drug concentration for each compound in retinal organoids. For that purpose, an LDH assay, determining the drug cytotoxicity, was used in combination with a range of different drug concentrations, selected for each drug individually, on retinal organoids (Supplementary Fig. S1A-G). As expected, ketorolac exposure was non-cytotoxic for retinal organoids and a concentration of 2.5 mM was chosen for further experiments (Supplementary Fig. S1A). Digoxin and thioridazine indicated dose-dependent cytotoxicity effects on retinal organoids and a concentration of 40 nM was used for digoxin and 135 µM for thioridazine in later experiments (Supplementary Fig. S1B, C). Sildenafil, ethanol, and methanol displayed the opposite, showing almost no toxic effects on retinal organoids after 24 h exposure (Supplementary Fig. S1D-G). Because sildenafil-related side-effects occurred after long-term use^29,56^ incubation was changed to 7 days, resulting in a final concentration of 225 µM (Supplementary Fig. S1D). For ethanol and methanol, we screened by IF the effect of different concentrations on photoreceptors (Recoverin) and RGCs (HuC/D), indicating some disruptive structural changes in the putative ONL (Supplementary Fig. S1F). Ethanol was used at a concentration of 500 mM (Supplementary Fig. S1E) and methanol’s final concentration was 32 mM (Supplementary Fig. S1G).

#### Impact of drugs on retinal organoids

##### Photoreceptors

Retinal organoids in all conditions were characterized by a thick neuroepithelium at the apical edge, often associated with an RPE patch next to it (Supplementary Fig. S1H, before). While there was no obvious change observed after control, ketorolac, sildenafil, ethanol and methanol incubation, digoxin- and thioridazine-treated organoid neuroepithelium were significantly thinner (Supplementary Fig. S1H, I, after), indicating drug-related alterations of organoids integrity. Cytotoxicity assessed by the LDH assay showed a consistent drug-related effect regardless of cell line (Supplementary Fig. S1J).

**Figure 3.**
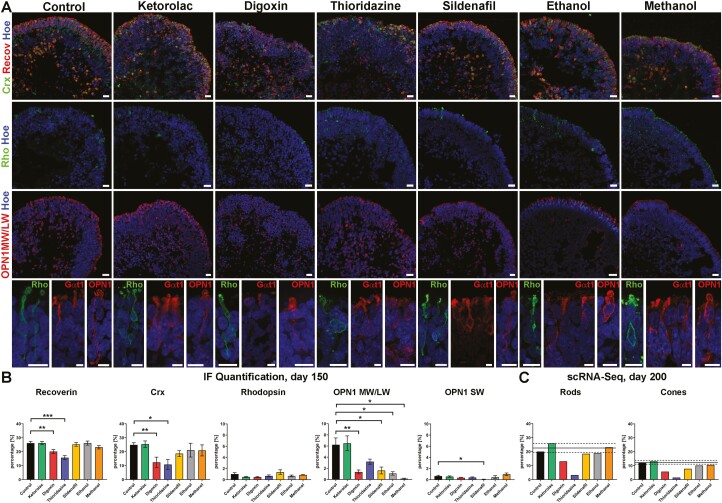
Impact of drugs on photoreceptors of retinal organoids derived from WT3 iPSCs. (**A**): Immunofluorescence of Crx (green), recoverin (Recov; red), rhodopsin (Rho; green), and OPN1MW/LW at day 150 of differentiation. Expression of Crx and recoverin was found at the apical edge of organoids in all conditions, except digoxin and thioridazine exposure where a disorganization of the photoreceptor layer was observed. Rods identified by rhodopsin immunofluorescence were rare in all retinal organoids at this time point of differentiation. Expression of OPN1MW/LW for middle and long-wavelength indicated mature and premature (nuclear staining) cones in all conditions. Higher magnification images revealed subcellular compartments of middle-/long-wavelength cones (OPN1; red) and rods (Rho; green) as well as rod OS (Gαt1; red). Nuclei were counterstained with Hoechst (blue). Scale bars, 20 μm (**B)**: Immunofluorescence quantification of recoverin, Crx, rhodopsin, OPN1MW/LW, and OPN1SW for all conditions. The exposure of digoxin and thioridazine resulted in a reduction of recoverin- and Crx-positive cells while rhodopsin expression was not affected after drug treatments at this time point of differentiation. OPN1MW/LW^+^ cells were reduced in all drug-treated conditions, except for Thioridazine whereas OPN1SW expression was affected only after sildenafil exposure. Data shown as mean ± SEM, *N* = 2 (independent experiments), and 5-20 images from different retinal organoids were quantified per condition. Differences were considered statistically significant at ∗*P* <.05, ∗∗*P* < .01 and ∗∗∗*P* <.001. (**C)**: Single-cell RNA-Seq data at day 200 of differentiation showed a reduction in the percentage of rods (clusters 0 and 7) after digoxin and thioridazine treatment, while the percentage of cones (clusters 2 and 19) decreased after digoxin, thioridazine, and sildenafil exposure. Data are shown as an individual percentage for each cell type and condition. The mean (black line) ± SEM (dashed line) of control and ketorolac conditions are shown. Hoe, Hoechst; Recov, Recoverin; Rho, Rhodopsin.

To investigate structural changes in retinal organoid organization and gene expression alterations in response to drug exposure, IF and scRNA-Seq analysis were performed at day 150 and day 200 of differentiation, respectively. Expression of the pan photoreceptor marker recoverin and the postmitotic photoreceptor marker CRX was observed at the apical edge of the organoids, forming a thick ONL in control and in sildenafil-, ethanol- and methanol-treated conditions (Fig. 3A). After treatment with digoxin and thioridazine, the ONL was disrupted, revealing a significant reduction of recoverin^+^ and Crx^+^ cells (Fig. 3A, B). These findings were also confirmed in retinal organoids derived from WT4 (Supplementary Figs. S2A, B and S3A, B). To gain more insights, IF with specific mature photoreceptor marker proteins rhodopsin for rods and red/green cone opsin (OPN1MW/LW) and blue cone opsin (OPN1SW), was carried out (Fig. 3A, B andSupplementary Fig. S4). Although rhodopsin expression was low (<2%; Fig. 3B), rhodopsin immuno-positive cells were found in the ONL across all organoids regardless of drug exposure (Fig. 3A, B). Higher magnification of selected rods (bottom images in Fig. 3A) revealed the morphology of a mature photoreceptor with cell body, axon, axon terminal, and inner/outer segment. Putative photoreceptor outer segments were additionally labeled with Gαt1 and found in all conditions (Fig. 3A, bottom images). The number of rhodopsin^+^ cells was unaffected by exposure to the various drugs at day 150 of differentiation (Fig. 3B); however, scRNA-Seq at day 200, showed a noticeable reduction in the percentage of Rhodopsin^+^ cells after digoxin and thioridazine treatment (Fig. 3C). This discrepancy may be due to the late onset of rhodopsin expression in organoids, where it usually peaks around day 180 of differentiation.^57^ Although we observed a higher percentage of rhodopsin^+^ cells in WT4 retinal organoids (Supplementary Figs. S2C and S3D), none of the drug treatments had an impact on rod presence at day 150 of differentiation.

For more detailed information about potential effects induced by drugs at the molecular level, differential expression gene analysis for all drug conditions compared with control was performed, followed by IPA analysis (Supplementary Tables S6 and S7). We observed changes in expression of key transcription factors involved in rod (*NR2E3*) and/or photoreceptor (*CRX*) specification in the digoxin- and thioridazine-treated groups, which may underlie and/or reflect the reduced rod presence in these 2 groups. These changes were accompanied by genes involved in critical pathways for photoreceptors such as the phototransduction pathway, signaling by Rho family GTPases, RhoGDI signaling, ephrin B signaling and CXCR4 signaling phototransduction (*GNGT1, NR2E3, RCVRN, CABP5, ROM1, RP1, NRL, CNGB1, KCNV2*), apoptosis and neuroprotection (*ASNS, MYOC, MT1G, TTR*),^58-60^ cell stress/photoreceptor survival (*IGFBP5*) and astrocyte remodeling (*GDF-15*^61^). The phototransduction pathway was also affected after sildenafil treatment, resulting in gene expression changes *(GNGT1, RGS16*). Notably, PDGF-AA, which can induce astrocyte activation and gliosis and target genes such as *CRYAB, S100A10, SOX2, GFAP, METRN*^62-68^ was identified as an upstream regulator. Methanol exposure had an impact on multiple pathways such as phototransduction pathway, IGF-signaling, notch signaling, Wnt/Ca^+^ pathway, and tRNA splicing. *CRX* was highlighted as an upstream regulator, affecting several genes including *IGFBP2*, *HES1*, *CLU*, *PTGDS*, *C1orf61* (all upregulated), and *PDE6A*, *RP1* (both downregulated). Ketorolac-treated organoids also demonstrated changes in expression of target genes associated with apoptotic and cell developmental functions (*ASNS*), neuroprotection (*TTR*^59^), photoreceptor survival (*IGBBP5*), rod (*NR2E3)*, and photoreceptor specification (*PNR*). Together these data may suggest a mild downstream effect of ketorolac in rod maturation.

Red/green cones expression (OPN1MW/LW) was found in retinal organoids in all conditions, although a mixed pattern of mature and immature red/green cone photoreceptor (the latter showing nuclear staining) was observed (Fig. 3A and Supplementary Fig. 2D). Higher magnification of red/green cones displayed the characteristics of photoreceptor cells (Fig. 3A). After drug exposure, the percentage of OPN1MW/LW^+^ cells decreased in all conditions (Fig. 3B). Blue cones (OPN1SW^+^) were found only rarely in organoids and no changes after drug treatment were observed, except for sildenafil (Fig. 3B and Supplementary Fig. D4). WT4 retinal organoids showed similar low expression of OPN1SW (below 1%; Supplementary Fig. S2E), failing to reveal any significant differences after drug treatment, except for thioridazine (Supplementary Fig. S3F). In accordance with the IF findings, the scRNA-Seq data revealed a decrease in the percentage of cone photoreceptors at day 200 of differentiation. This was more prominent after digoxin, thioridazine, and sildenafil treatment than after ethanol and methanol exposure (Fig. 3C). Differential gene expression analysis for digoxin treated organoids revealed *GNB3* (a gene essential in phototransduction and ON-bipolar cell signaling^69^) as an upstream regulator, targeting a network associated with cell signaling, molecular transport, and nucleic acid metabolism where *PCP2* is downregulated and *LRATD1* is upregulated (Supplementary Tables S6 and S7). Thioridazine also had an impact on the phototransduction pathway in rod photoreceptors, revealing a downregulation of essential genes such as *PDE6G, PHE6H, GNB3, GUCA1A, CNGB1, GUK1, GNAT1, GNGT1,* and *GNGT2* (Supplementary Tables S6 and S7). Further, affected genes included *TRPM1*, *AQP1*, *GDF-15,* which are involved in a network associated with cell death and survival, and cell cycle. Sildenafil treatment induced the upregulation of *CRYAB* and *VIM* functioning in the 14-3-3 mediated signaling, which is associated with cell death and survival.

Together these data indicate that drug treatments result in phenotypic changes, indicating that cones were more affected than rods at day 150 of differentiation. At the later stages of differentiation (day 200) changes in the expression of genes that play important roles in photoreceptor specification, phototransduction, cell stress, and apoptosis-related pathways were noted, reflecting the photoreceptor cell-specific response to these drugs.

##### I nterneurons

**Figure 4.**
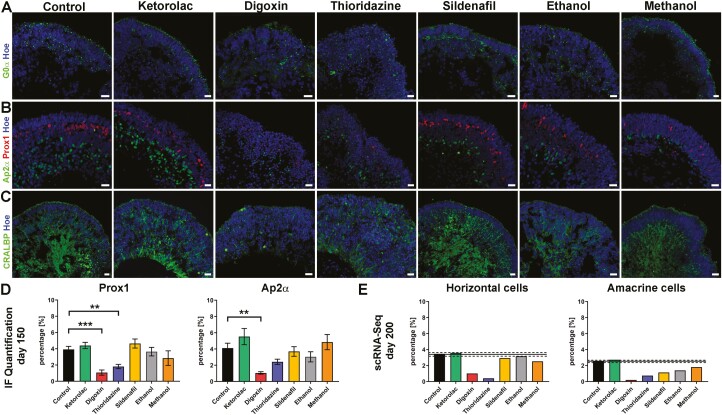
Drug effects on bipolar, horizontal, amacrine, and Müller cells of WT3-derived retinal organoids. (**A-C)**: Expression of bipolar cells (G0α; green), horizonal cells (Prox1; red), amacrine cells (Ap2α; green) at day 150 of differentiation. Expression of bipolar marker G0α was found in all retinal organoids, even after drug treatment (**A**). Prox1-positive horizontal cells were located closer to the photoreceptor layer than amacrine cells, which were more distal in the organoids (**B**). Prox1-positive cells were disorganized after thioridazine and digoxin exposure; the same was observed for Ap2α-positive cells only after digoxin incubation (**B**). Müller glia cells stretched through the whole neuroepithelium of organoids (**C**). Their organization was affected in digoxin and thioridazine-treated organoids, showing a total disruption of Müller glia cells after digoxin exposure. Nuclei were counterstained with Hoechst (blue). Scale bars, 20 μm (**D)**: Immunofluorescence quantification of Prox1 and Ap2α for all conditions, revealed a decline in Prox1 and Ap2α percentage after digoxin exposure, while thioridazine treatment resulted in a reduction of Prox1-positive cells only. Data shown as mean ± SEM, *N* = 2 (independent experiments), and 5-12 images from different organoids were quantified per condition. Differences were considered statistically significant at ∗∗*P* <.01 and ∗∗∗*P* <.001. (**E)**: Single-cell RNA-Seq data for horizontal and amacrine at day 200 of differentiation, indicated a downregulation in the percentage of horizontal and amacrine cells (horizontal cell cluster. 13; amacrine cell 18). Data are shown as an individual percentage for each cell type and condition. The mean (black line) ± SEM (dashed line) of control and Ketorolac conditions are shown. Hoe, Hoechst.

The INL of retinal organoids contained bipolar cells, labeled with G0α (a marker for Rod- and some ON Cone bipolar cell types), HCs identified by Prox1, Ap2α-positive ACs, and MCs stained for CRALBP (Fig. 4A-C and Supplementary Fig. S5A-C). The percentage of HCs decreased after digoxin and thioridazine exposure at day 150 of differentiation (Fig. 4B, D and Supplementary Fig. S5D). This was confirmed by scRNA-Seq at day 200 of differentiation (Fig. 4E). The percentage of Ap2α^+^ ACs was reduced after Digoxin exposure at both day 150 and 200 of differentiation (Fig. 4B, D, E and Supplementary Fig. S5A, D). While MCs were not affected in the control, ketorolac-, sildenafil-, ethanol-, and methanol-treated groups, Thioridazine treatment resulted in disorganization and digoxin exposure even led to a disruption of MCs (Fig. 4C and Supplementary Fig. S5C). The IPA analysis revealed activation of the unfolded protein response pathway in response to thioridazine treatment, as indicated by the upregulation of several typical genes (eg, *CEBPD, HSPA5, HSPA6,* and *DDIT3*) (Supplementary Tables S6 and S7). After digoxin exposure, another pathway was highlighted namely the HIF1 signaling pathway. It was shown that digoxin can inhibit HIF1α synthesis, inducing cell-cycle arrest and apoptosis^[18]^, which may explain the disruption of MCs seen by IF at day 150 of differentiation.

##### Retinal ganglion cells

**Figure 5.**
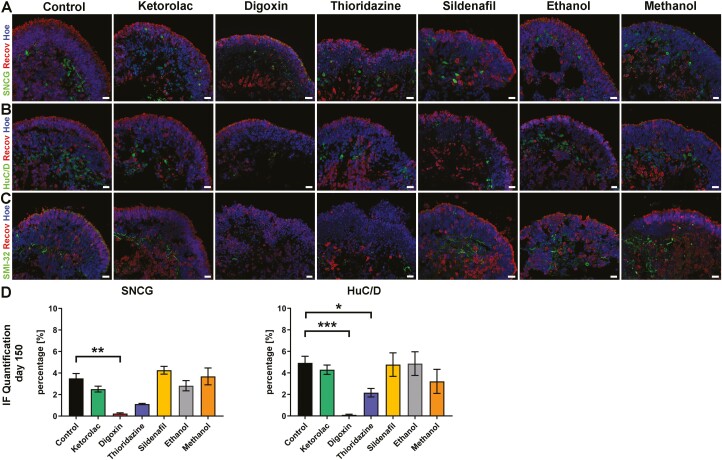
Drug impacts on retinal ganglion cells of WT3-derived retinal organoids. **(A-C)**: Expression of SNCG (**A**), HuC/D (**B**), and SMI-32 (**C**) identifying retinal ganglion cells. Photoreceptors are immunostained with recoverin (**A-C**). SNCG- (green; **A**) and HuC/D-positive cells (green, **B**) and SMI-32 immunofluorescence positive dendrites/axons were found in the center of retinal organoids while photoreceptors (Recov, red; **A-C**) formed a layer at the apical edge of retinal organoids at day 150 of differentiation. Nuclei were counterstained with Hoechst (blue). Scale bars, 20 μm. (**D)**: Immunofluorescence quantification of SNCG and HuC/D for all conditions, showing a significant reduction of HuC/D-positive cells after digoxin and thioridazine exposure, while this effect for SNCG was only seen after digoxin treatment. Data are shown as mean ± SEM, *N* = 2 (independent experiments), and 5-20 images from different organoids were quantified per condition. Differences were considered statistically significant at ∗*P* < .05, ∗∗*P* < .01 and ∗∗∗*P* <.001. Hoe, Hoechst; Recov, recoverin.

RGCs formed the third layer of the retina (the GCL) and were found in the center of the organoids visualized by IF using different marker proteins such as SNCG, an RGC marker protein, HuC/D, and SMI-32, both labeling RGCs and specific subpopulations of ACs (Fig. 5). SNCG- and HuC/D-positive RGCs were seen in both control and in all drug conditions, except for digoxin, where very few immuno-positive cells could be found (Fig. 5A, B and Supplementary Fig. S2B). Thioridazine exposure also resulted in a significant reduction of HuC/D-positive cells (Fig. 5D). Correspondingly, this effect was reflected in immuno-positive cells for SNCG, although the data showed a statistical trend (*P* = .057; Fig. 5D) rather than significance. The decline of SNCG-positive RGCs was also noticeable after digoxin and thioridazine treatment in retinal organoids derived from the second hiPSC line, WT4 (Supplementary Figs. S2B and S3C). These findings were corroborated by SMI-32 IF data (Fig. 5C).

IPA analyses of the RGC cell subsets after thioridazine treatment highlighted the antiproliferative role of somatostatin receptor 2 pathways, showing a downregulation of somatostatin (*SST*) and cell cycle: G2/M DNA damage checkpoint regulation concomitant with the p53 signaling, where *RPRM* (downregulated) is involved as the main target gene (Supplementary Tables S6 and S7). Within this network, additional genes including *ID3, PROX1* (both upregulated)*, LRRN3, OLFM3, CPLX3, and RCVRN* (all downregulated) were affected. It is interesting to note that *LRRN3* and *CPLX3* are linked with the regulation of synapses and or synapse assembly.^70,71^ Axonal guidance signaling and semaphorin neuronal repulsive signaling pathway were prominent in the analysis of the differentially expressed genes for digoxin. It has been reported that *SEMA3*s are involved in RGCs apoptosis during development as well as playing an important role in regulating synaptic plasticity in the mature retina.^72^ Hence, SEMA3C (upstream regulator) signaling through *PLXNA2* (which is upregulated) may lead to cell death as shown by our IF data.

**Figure 6.**
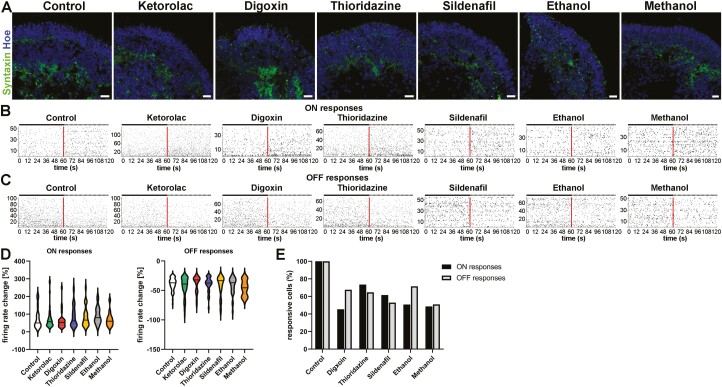
**Drug effects on the physiological function of retinal organoids derived from WT3 iPSCs**. (**A)**: Syntaxin expression (green) was observed in retinal organoids putative OPL and IPL, showing less organized layer-like expression after ethanol exposure. Nuclei were counterstained with Hoechst (blue). Scale bars, 20 μm. (**B, C)**: Spike raster plots from putative ON-center retinal ganglion cells (RGCs; **B**) and Off-center RGCs (**C**) of WT3-derived retinal indicated an increase in spiking activity for ON-center like RGCS and a decline in their spiking activity for Off-center like RGCs after white light pulses (WLP). Each row represents a different RGC, and each vertical bar represents a spike from the corresponding RGC. The red line illustrates the stimulus onset whereas the left half before indicates the spontaneous (baseline) activity before WLP exposure and the right half when exposed to WLP. (**D)**: Violin plots show the percentage (%) firing rate change of putative ON-RGCs and OFF-RGCs after WPL, revealing no significant differences (Mann-Whitney test). Data are represented by the median and interquartile ranges with Tukey whiskers. (**E)**: Number of active RGCs (in %) for all drug conditions in relation to control groups (control and ketorolac) indicated less spiking RGCs for ON-center- and Off-center-like responses after WPL in all drug-treated retinal organoids.

In the ethanol-treated group, the IPA analysis revealed a role for the synaptogenesis signaling pathway, targeting the *SYN4* gene, which was downregulated. This suggests that ethanol may influence synaptogenesis, corroborating the Syntaxin IF data at day 150 of differentiation (Fig. 6A) and previous studies.^38,43^ Like thioridazine, after ketorolac treatment, differentially gene expression analysis emphasized the antiproliferative role of somatostatin receptor2 pathway together with BAG2 signaling and unfolded protein response pathway, indicating an upregulation of *HSPA6* and a downregulation of *SST*. This suggests that ketorolac, despite the lack of structural changes in our study (Fig. 5) and published ophthalmic side effects, can influence the gene expression pattern in RGCs.

In addition to gene expression changes, physiological functionality was tested using RGC MEA. Immunostaining of Syntaxin, a presynaptic plasma membrane marker protein, confirmed that connections between retinal cell types were taking place in the putative OPL and IPL (Fig. 6A) which is essential for physiological assessment of the organoids in addition to the expression of all retinal cell types. MEA was performed at day 150 of differentiation and RGC responses upon full-field white light pulses (WPL) were analyzed separately for ON-center- and Off-center-like RGC responses (Fig. 6B). RGC activity after WPL indicated either an increase for ON-center-like responses or a decrease for Off-center-like responses in control conditions. Additionally, no changes in responsiveness to light were detected for any drug-treated condition (Fig. 6B) and, there was no significant difference in the percentage of firing rate change for either RGC ON-center- or Off-center-like responses in drug conditions when compared to control (Fig. 6C). Drug exposure (except for Ketorolac as a control drug) resulted in a decrease in the number of light-responsive RGCs, notable for both ON-center- and Off-center-like responses (Fig. 6D). These findings suggest that although light response patterns are not affected, fewer cells are able to respond to light following treatment with all the drugs used in this study, which corroborates IF and scRNA-Seq data.

### Drug Exposure Leads to Apoptosis and Astrocyte Activation

To further validate the observed reduction of diverse retinal cell types upon drug treatment, Caspase-3, a key marker for apoptosis, IF was performed (Supplementary Fig. S6). An increase in Casp-3^+^ cells was found after digoxin, thioridazine, and ethanol exposure in retinal organoids derived from WT3 and WT4 (Supplementary Fig. S6). Thus apoptosis, mediated by caspases, may be the underlying mechanism for cell death after drug treatment in some conditions.

**Figure 7.**
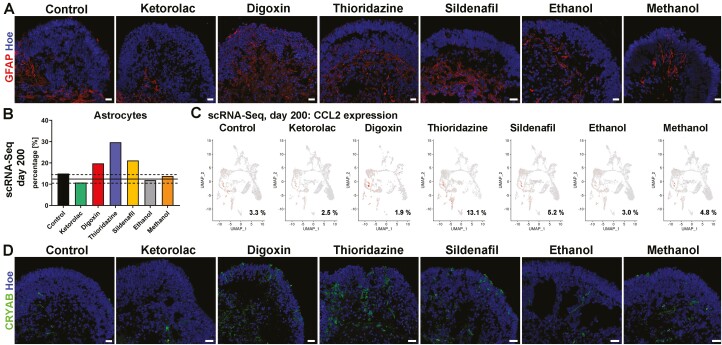
Astrocyte activation in WT3 derived retinal organoids after drug treatment. (**A)**: Astrocytes (GFAP; red) were observed in all retinal organoids across all conditions. While astrocytes were found underneath the neuroepithelium in the center of retinal organoids in control and ketorolac condition they sprouted out into the neuroepithelium after exposure to digoxin, thioridazine, sildenafil, ethanol, and methanol, suggesting activation of astrocytes. Nuclei were counterstained with Hoechst (blue). Scale bars, 20 μm. (**B)**: A higher percentage of astrocytes was found in retinal organoids treated with digoxin, thioridazine, and dildenafil. Data are shown as an individual percentage for each cell type and condition. The mean (black line) ± SEM (dashed line) of control and ketorolac conditions are shown. (**C)**: *CCL2* gene expression (shown on the original UMAP) revealed an increase in the percentage of expressing cells upon thioridazine treatment. (**D)**: An increase in the fraction of cells expressing α-crystallin B (CRYAB; green) expression was noted after digoxin, thioridazine, and sildenafil exposure. Nuclei were counterstained with Hoechst (blue). Scale bars, 20 μm. Hoe, Hoechst.

Astrocytes, non-retinal glial cells, are mainly found in the nerve fiber layer and GCL of the retina and enter the retina during the development of the optic nerve.^73^ Retinal organoids also contain astrocytes, as indicated by glial fibrillary acidic protein (GFAP) staining on day 150 and scRNA-Seq at day 200 of differentiation (Figs. 7 and 2A). These putative astrocytes were found in the center of the organoids, mostly restricted to the putative GCL in control and ketorolac conditions, concluding that GFAP^+^ labeled cells are astrocytes rather than active MCs. After drug exposure, these GFAP^+^ cells additionally spread out into retinal layers (Fig. 7A). IF analyses suggested an increase in the number of astrocytes after some drug treatments such as digoxin and thioridazine. Remarkably, this effect was also shown by the scRNA-Seq analysis, revealing an upregulation in astrocytes within digoxin, thioridazine and sildenafil-treated groups when compared to the control conditions (Fig. 7B). Differentially expressed gene analysis indicated canonical pathways such as the unfolded protein response, the endoplasmic reticulum stress pathway, IL-12/17 signaling, the glutathione redox reactions I and the HIF1 signaling to be affected after drug exposure, involving upregulation of *HSPA6*, *APOE*, *APOC1*, *CCL2*, *CXCL2*, and *CXCL3* as well as downregulation of *HSP90B1*, *XBP1*, *CLU,* and *MGST1* (Supplementary Table S6 and Table 7). A notable increase in the percentage of overall cells (and astrocytes) expressing *CCL2*, which is triggered and released during inflammatory processes was observed after thioridazine treatment (Figure 7C), indicating the possibility of astrocyte activation. Additionally, the expression α-crystallin (CRYAB) which is known to protect cells from stress by binding misfolded proteins and/or inhibiting apoptosis was noted in many more cells after digoxin and thioridazine exposure (Figure 7C). Interestingly, CRYAB upregulation in astrocytes and Müller glia cells has been linked with several disease-associated neuroinflammation models and diabetes rodent models, suggesting a role in the survival of cells or in activation of glia cells.^63^ Together with IF data, showing sprouting of astrocytes into retinal neuroepithelium, these findings suggest that the drug exposure results in cellular stress with an inflammation-like response, which may lead to astrocyte activation and/or remodeling as shown in animal studies associated with the retinal diseases.^68,74,75^

## Discussion

This comprehensive study provides novel insights in toxicological drug testing, exposing retinal organoids derived from 2 different hiPSCs to well-known drugs. Retinal organoids generated in this study resembled the human retina morphologically, displaying the typical 3 nuclear-layered structure, interspersed with 2 plexiform layers, and containing all retinal cell types, with photoreceptors comprising IS, connecting cilium and OS. Remarkably, RGCs were preserved for the whole length of differentiation, and further specified into different subsets as demonstrated by Tran et al.^55^ Likewise, bipolar cells also developed into different subtypes, providing further evidence of similarity to the human retina. To this end, we used these retinal organoids to test the impact of several well-known retinotoxic agents. Our data indicate that ketorolac was nontoxic to retinal organoids, displaying no changes by IF at day 150 of differentiation when compared to the control group. Digoxin and thioridazine were the most potent retinal cytotoxic compounds, causing a reduction of photoreceptors, horizontal, amacrine, Müller glia, and retinal ganglion cells, whereas sildenafil mainly affected photoreceptors. In addition, astrocyte sprouting into the neuroepithelium was obvious after all drug treatments. All retinal organoids were functional, revealing light responsiveness, even so, although fewer RGCs did respond to after drug treatment compared to the control groups.

### Drug Effects of Retinal Organoids Are Comparable to In Vivo Responses

Retinal organoids treated with ketorolac were similar to vehicle-treated control organoids with regard to morphology, structure/organization, and functionality. This nontoxic effect on the retina is in accordance with the previous literature.^14,15,76-78^ Notwithstanding, our gene expression analysis indicated changes in gene expression including the upregulation of Heat Shock Protein Family A (Hsp70) Member 6 (*HSPA6*) in astrocytes and RGCs. *HSPA6* is involved in the “unfolded protein response pathway (UPR)” and plays an essential role in the correct folding and/or refolding of misfolded proteins as well as directing proteins for degradation. A possible reason for the change in the gene expression pattern of RGCs and astrocytes may result from ketorolac’s inhibitory effects of COX enzymes which are expressed by RGCs, displaced ACs, astrocytes, and microglia in the human retina.^79^ Despite being known that overall NSAIDs can induce the UPR and subsequentially apoptosis in various cell systems,^80-82^ this is the first report of a ketorolac-induced retina related effect, which emphasizes the importance of scRNA-Seq as a useful method to unveil early drug-related changes in gene expression level rather than later changes detectable using anatomical/functional assays, at a time when changes may be nonreversible eg when cells are dying.

Treatment with digoxin caused multiple damages to retinal organoids including cell death of photoreceptors, horizontal, amacrine, and ganglion-cell death as well as a disruption of Müller glia cells. Furthermore, digoxin activated stress and apoptosis signaling pathways and induced genes involved and/or associated with retinal degeneration and inflammation in the retinal organoids. It has been shown that digoxin causes ocular symptoms, arising mainly from photoreceptor alterations and/or damage after exposure, and the activation of cellular stress signaling (Table 1). Our data do indeed corroborate the impact of digoxin on photoreceptors, but moreover also reveal drug-specific effects in all retinal cell types, except for bipolar cells. A possible reason for these broad effects may be explained by digoxin’s inhibitory effect on Na^+^/K^+^ ATPase ion pumps. The catalytic subunit alpha (α1-3) of Na^+^/K^+^ ATPase pumps are the primary target of digoxin^83,84^ which are widely expressed across the retina.^85^ Subunit α1 is expressed in several retinal cell types, while subunit α2 is restricted to Müller cells and subunit α3 is present in all retinal cell types, except for Müller glia cells and RPE.^84,85^ The inhibition of the Na^+^/K^+^ ATPase pump leads to an imbalance of the transmembrane ion gradients with an increase of Na^+^ and a decrease of intracellular K^+^ concentration. Yu and colleagues^86^ demonstrated that disruption of K^+^ homeostasis can initiate apoptosis in cells and photoreceptor degeneration in fruit flies lacking the Na^+^/K^+^ ATPase alpha subunit.^87^ This may suggest that alteration of intracellular K^+^ homeostasis upon digoxin application could be the underlying reason for the cell death observed. Furthermore, it has been shown that digoxin application has an inhibitory effect on the HIF1 signaling pathway,^74,88,89^ and can inhibit tumor growth by inducing cell-cycle arrest and apoptosis.^89-91^ Our scRNA-Seq data also indicate an involvement of the HIF1 pathway, adding hypoxic conditions as a possible reason for cell death upon digoxin application. To the end of this, it is important to note that also the application method of drugs can influence the drug impact on the tissue of interest. Digoxin (and all other drugs in this study) was administered to the culture medium, enabling direct exposure of retinal organoids to the drug, unlike reported human case studies and/or most in vivo animal toxicological studies where the drug is not directly administered to the tissue of interest, the eye. This may result in more profound changes in both cell survival and gene expression as revealed by our study.

Similarly, to digoxin, thioridazine exposure caused significant damage to retinal organoids. Disorganization and/or loss of photoreceptor outer segments followed by RPE loss has been reported in human patients,^92^ which is in line with our IF findings at day 150 of differentiation. Additionally, the phototransduction pathway was significantly impacted. A plausible explanation for this could be thioridazine’s suggested underlying mode of action, namely the alteration of enzyme kinetics, which leads to inhibition of oxidative phosphorylation followed by abnormal rhodopsin synthesis.^93^ In accordance with these findings, thioridazine exposure caused upregulation of several genes (eg, *GDF-15, MT1G, CLU, XBP1, HSPA6, HSPA5, CCL2, CXCL3*, and *CXCL2*) encoding proteins associated with oxidative stress, inflammation, and Müller glia cell and/or astrocyte activation/remodeling in retinal organoids.^61,68^

Retinal organoids exposed to sildenafil displayed mainly photoreceptor alterations, with a pronounced impact on cone photoreceptors compared to rods. These findings follow reported sildenafil effects in humans and in in vivo studies, demonstrating that photoreceptors are the primary affected cell type (Table 1), and reporting ocular symptoms such as impairment in color vision and light perception, blurred vision, photophobia, and transient changes in the ERG (Table 1). Sildenafil inhibits largely PDE5, which is expressed in bipolar and RGCs of the human retina.^94^ More importantly, sildenafil also affects PDE6, which is expressed in rods and cones, differing slightly in its catalytic subunit composition.^95^ It plays a major part in the phototransduction cascade where its activation is triggered by a G protein, transducin, upon photon absorption. A high intracellular cGMP concentration prevalent in the dark is reduced by PDE6, hydrolyzing cGMP to GMP. This results in a closure of CNG cation channels in the membrane of the photoreceptors and subsequently to membrane hyperpolarization in photoreceptors.^96^ It was shown that elevation of intracellular cGMP levels either by blocking PDE6 or inducing a mutation in the *PDE6* gene which is involved in a subset of human RP cases leads to photoreceptor apoptosis and retinal degeneration.^97-100^ Therefore, disrupting the intracellular cGMP level and subsequently ion homeostasis upon sildenafil exposure may cause a negative effect on photoreceptors, associated with upregulation of photoreceptor-related genes involved in the phototransduction pathway.

Most published studies reporting ocular cytotoxicity in humans are based on oral drug administration, preventing an exact knowledge of drug concentrations that reach and are metabolized in the retina. Our dose range studies were mostly informed by the local administration of these drugs in animal studies. Notwithstanding this caveat, similar responses to those observed in vivo in human reports were found with organoids, indicating the usefulness of organoid-based platforms for toxicology studies.

### Common Drug Effects and Their Impact for Toxicological Studies in the Future

This study investigated drug effects on retinal organoids derived from two different hiPSC lines to validate the robustness of retinal organoids as model systems for drug and toxicology studies. Variability of retinal organoids is a known issue in the field, however, to overcome this we used both IF and single-cell RNA-Seq methods to corroborate our results. Importantly, this study compared drug effects of two different hiPSC-derived retinal organoids, demonstrating similar results in both cell lines examined by LDH assay, IF, and functional recordings. For example, the results of the initially assessed cytotoxicity of each drug differed between 5-10% from retinal organoids derived from both cell lines. This acceptable range emphasizes the cell line independent drug effects and the robustness of retinal organoids as an *in vitro* model.

A common feature upon drug exposure was the activation of inflammatory and stress-induced pathways as well as the Müller glia cells and/or astrocyte proliferation in which the following genes were often changed: *CD24*, *C9orf24*, *AQ1*, *SST*, *TTR*, *CRYAB*, *GDF-15*, *APOE,* and *HSPA6*. Differential gene expression analysis of astrocytes showed a downregulation in gene expression of *CD24* and *C9orf24* upon all drug treatments. The latter is associated with differentiation and function of ciliated bronchial epithelial cells, possibly playing an important role in ciliogenesis, while Cluster of Differentiation 24 (*CD24*), a small glycosyl phosphatidylinositol (GPI)-linked cell surface glycoprotein is ubiquitously expressed in humans and often used as a marker for hematopoietic and neuronal cell differentiation.^101^ Lately, CD24 was also implicated as a novel biomarker in diagnosis and prognosis of several cancer types,^101,102^ showing that CD24 expression is correlated with tumor proliferation, invasiveness, and metastasis and subsequently with poor prognosis in, eg, breast cancer and small cell lung carcinomas.^101,103-105^ Hence, drug-induced changes in CD24 expression indicate that CD24 may provide a novel biomarker to assess retinal cytotoxicity associated with stress-induced activation of astrocytes. Further upregulation of *CRYAB, GDF-15*, and *AQ1* in photoreceptor cells as well as of *HSPA6* and *SST* in RGCs were observed after drug treatment. Like *CD24*, some of these markers are already used as predictive biomarkers for certain types of cancer such as *CRYAB*,^106^*HSPA6*,^107,108^, *GDF-15*^109,110^, and somatostatin (*SST*)^111^ and thus may offer potential biomarkers to predict drug-related effects in retinal organoids. Importantly, changes in expression of a key photoreceptor-specific gene, namely *NE2E3*, and a protein of the interphotoreceptor matrix, *IGFBP5* which was seen after digoxin, thioridazine, and ketorolac treatment, suggest that these genes may be suitable to monitor drug-induced effects on photoreceptors. It is worthwhile noting that all these highlighted genes may provide putative predictive biomarkers for retinal cytotoxicity studies, but this needs to be addressed in future work.

Retinal function was assessed by MEA recordings, showing fewer light-responsive RGCs in drug-treated compared to control organoids and indicating that the signaling pathway from photoreceptor to RGCs is affected upon drug exposure. This matches with drug-related effects on retinal organoids indicated by IF and scRNA Seq. However, it cannot be further linked with visual disturbances reported by patients (eg, changes in color vision), nor can it be associated directly with specific cell types eg, photoreceptors. To decipher these questions, other physiological methods such as electroretinograms, patch-clamp recordings, and/or calcium imaging should be considered in future studies.

## Conclusion

This comprehensive study provides new insights for large-scale drug screening by demonstrating that the retinal organoids offer a robust model for toxicological studies, displaying within a tolerable range comparable drug-induced effects to those reported from in vivo animal studies and human case reports. This first-time proof of principle study showcases the use of hPSC-derived retinal organoids for *in vitro* toxicological studies, enabling drug safety assessments and the development of new therapies in a wide range of diseases, including non-treatable retinal diseases, neurodegenerative disorders, and cancer with the benefit of reducing the number of in vivo animal experiments. Although the hPSC-derived retinal organoids do not comprise a specific cone enriched region akin to the human fovea, they do contain cone photoreceptors, which show drug-specific sensitivity. The methods and assays developed herein will provide a useful pipeline to further test “fovea” like organoids with retinotoxic drugs to get additional insights on their impact on the high-acuity region of the human retina.

## Supplementary Material

szab010_suppl_Supplementary_Material

szab010_suppl_Supplementary_Table_S2

szab010_suppl_Supplementary_Table_S3

szab010_suppl_Supplementary_Table_S4

szab010_suppl_Supplementary_Table_S5

szab010_suppl_Supplementary_Table_S6

szab010_suppl_Supplementary_Table_S7

## Data Availability

The data that support the findings of this study are openly available in GEO, reference number GSE172138.
