# Supplementary material for: Human Retinal Organoids Provide a Suitable Tool for Toxicological Investigations: A Comprehensive Validation Using Drugs and Compounds Affecting the Retina
Source: Stem Cells Transl Med. 2022 Feb 19;11(2):159–77. doi: 10.1093/stcltm/szab010 (PMC8929478; doi:10.1093/stcltm/szab010)
Supplement: szab010_suppl_Supplementary_Material [file szab010_suppl_supplementary_material.pdf]

Supplement Information

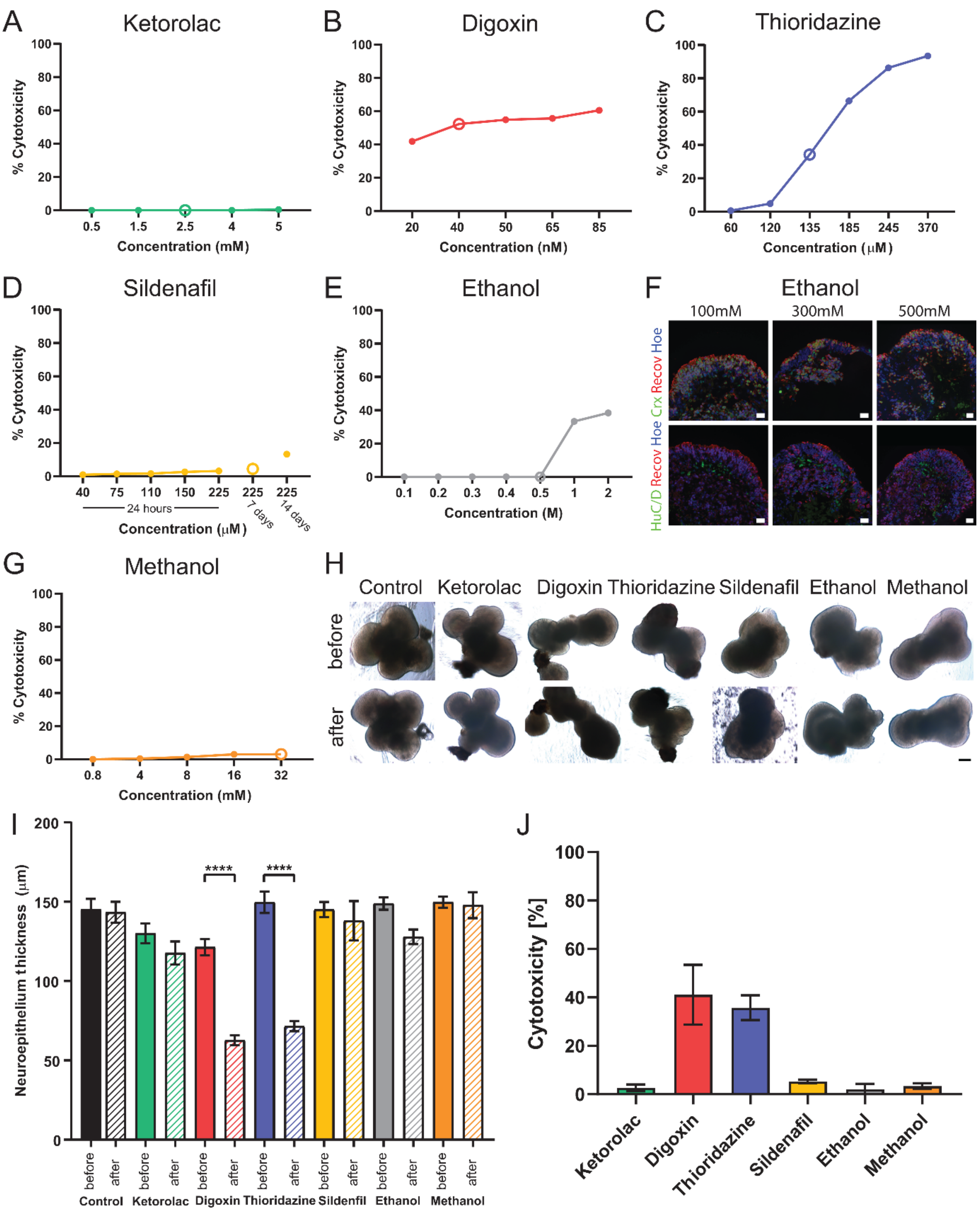

Fig. S1. Optimal drug concentration evaluation, drug cytotoxicity of drugs and morphological induced changes. A-G: Assessment of optimal drug concentration whereby each drug was tested using at least 5 different concentrations, shown for Ketorolac (**A**), Digoxin (**B**), Thioridazine (**C**), Sildenafil (**D**),

Ethanol (**E**) and Methanol (**G**). Non-filled circles denote the final drug concentration used for toxicological experiments. Immunofluorescence for Crx (green), Recoverin (Recov; red) and HuC/D (green) at different Ethanol concentrations revealed minor structural changes after 300 mM Ethanol exposure, which were more prominent in 500 mM Ethanol condition (**F**). Nuclei were counterstained with Hoechst (blue). Scale bars, 20 µm. **H:** Brightfield images before and after drug exposure at day 150 of differentiation indicated a thinning of neuroepithelium in Digoxin- and Thioridazine treated organoids. Scale bars, 20 µm. **I:** Neuroepithelium quantification revealed a thinning of the neuroepithelium upon Digoxin and Thioridazine treatment. Data are shown as mean ± SEM. Differences with a group (before versus after) were considered statistically significant at ****$p < 0.0001$. **J:** Elicited cytotoxicity on retinal organoids after optimal drug exposure at day 150 of differentiation. Data are represented as mean (WT3 and WT4 pooled) ± SEM, N=3. Abbreviations: Hoe, Hoechst.

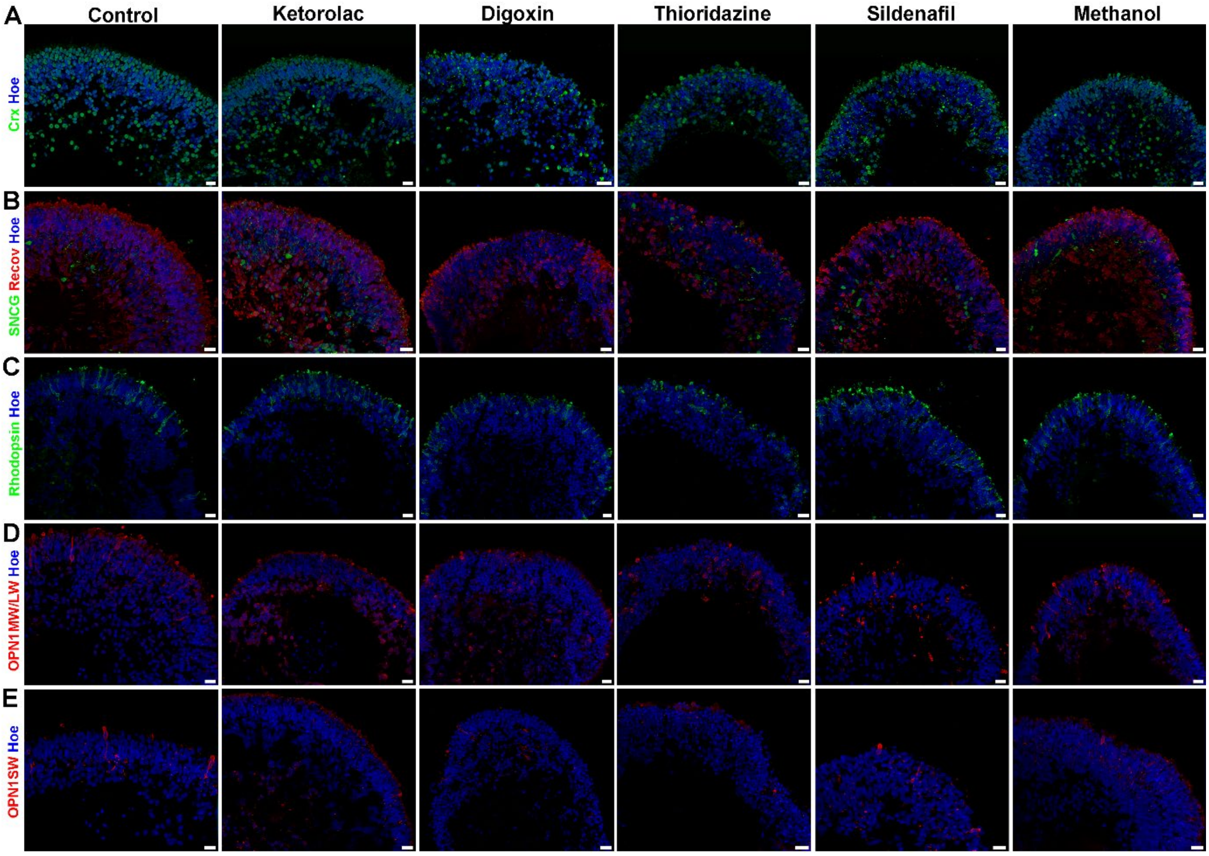

Fig. S2. Impact of drug treatment on photoreceptors and retinal ganglion cells in WT4 organoids after drug exposure. **A-E:** Expression of Crx (**A**), Recoverin (**B**), HuC/D (**B**), Rhodopsin (**C**), OPN1MW/LW (**D**) and OPN1SW (**E**) at day 150 of differentiation. Crx (green; **A**), Recoverin (red; **B**), Rhodopsin (green; **C**), OPN1MW/LW (red; **D**) and OPN1SW (red; **E**) immunoreactivity was observed at the apical edge of retinal organoids, revealing changes after Digoxin and Thioridazine treatment compared to control organoids. HuC/D-positive retinal ganglion cells (green) were found in the center of retinal organoids. Digoxin and Thioridazine exposure affected HuC/D expression negatively (**B**). Nuclei were counterstained with Hoechst (blue). Scale bars, 20 µm. Abbreviations: Hoe, Hoechst; Recov, Recoverin.

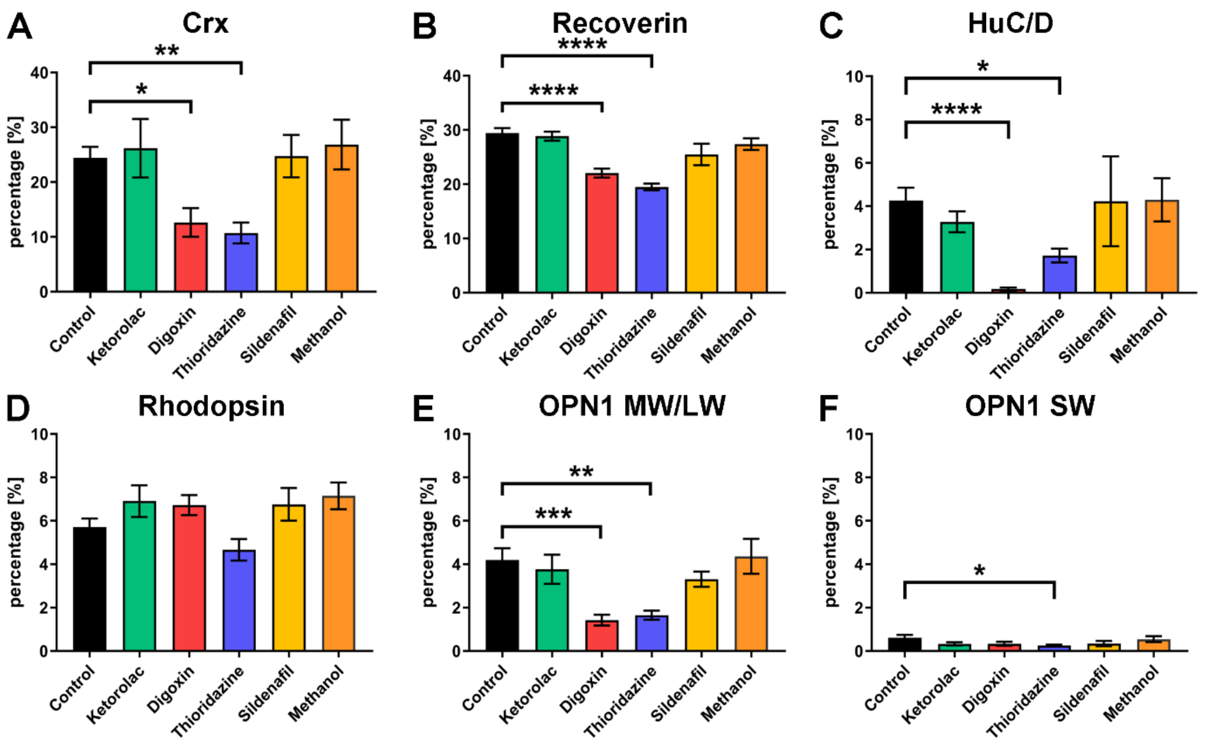

Fig. S3. Immunofluorescence quantification of retinal organoids derived from WT4-iPSCs at day 150 of differentiation. **A-F:** Significantly fewer positive cells for Crx (**A**), Recoverin (**B**), HuC/D (**C**), OPN1MW/LW were detected in Digoxin and Thioridazine treated organoids, whereas a significant reduction in OPN1SW-positive cells (**F**) was seen only after Thioridazine exposure. Rhodopsin expression was not significantly different across all conditions (**D**). Data represents the mean ± SEM, of 5-10 images from different organoids were quantified per condition. Differences were considered statistically significant at *p < 0.05, **p < 0.01, ***p < 0.001 and ****p < 0.0001. Please note, IF analysis for Ethanol group was not possible due to sections being fragile.

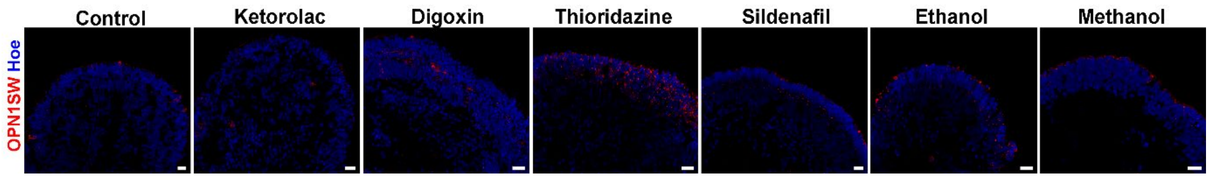

Fig. S4. Expression of short wavelength opsin in retinal organoids derived from WT3-retinal organoids at day 150 of differentiation. Expression of OPN1SW (red) at the organoid's apical edge and was less prominent after Sildenafil exposure. Nuclei were counterstained with Hoechst (blue). Scale bars, 20 µm. Abbreviations: Hoe, Hoechst.

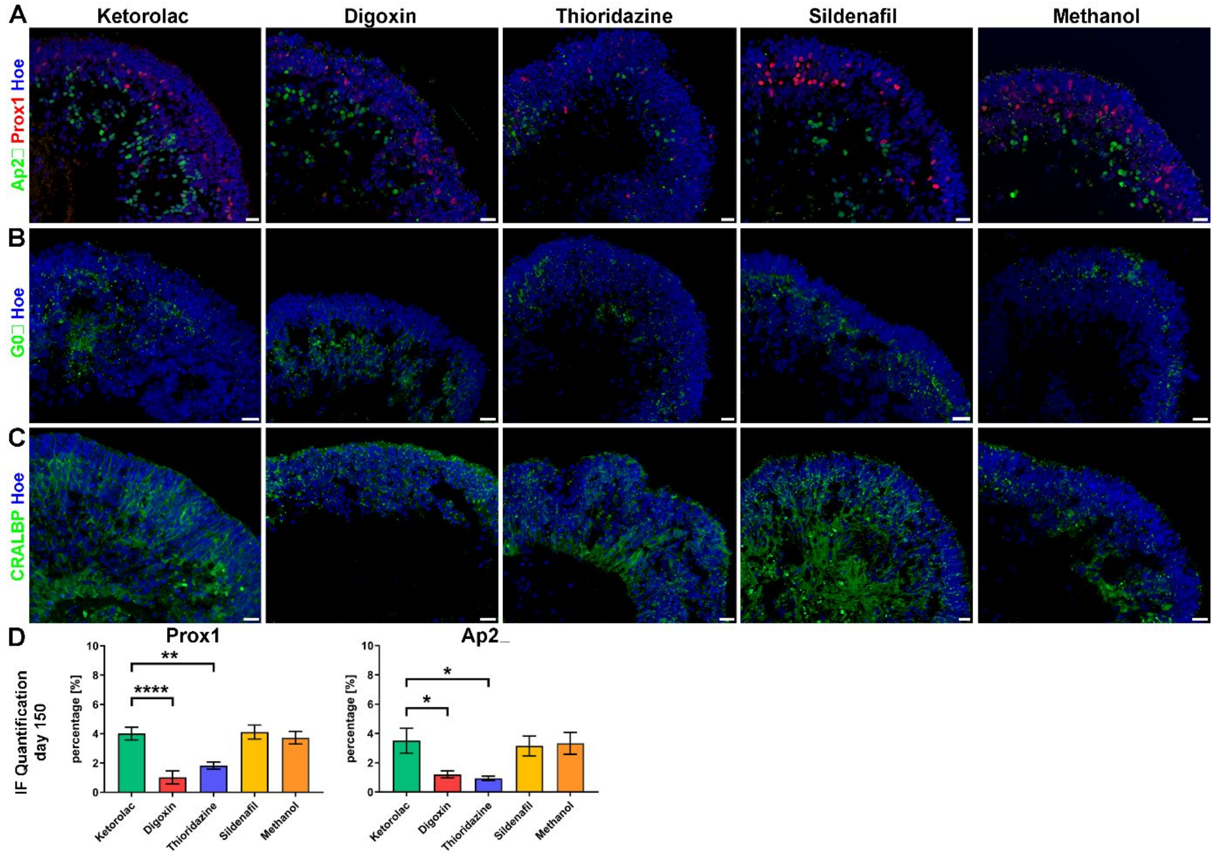

Fig. S5. Drug effects on bipolar, horizontal, amacrine and Müller glial cells in WT4- derived retinal organoids. A-C: Expression of horizonal (Prox1; red), amacrine (Ap2α; green), bipolar (G0α; green) and Müller glia cells at day 150 of differentiation. Horizontal (Prox1; red) and amacrine cell (Ap2α; green) were less organized after Digoxin and Thioridazine exposure compared to retinal organoids in the drug control condition, Ketorolac (**A**). Bipolar cells (G0α; green) were found in all conditions, even so the expression of this marker was low (**B**). Müller glia cells were disrupted and/or disorganized after Digoxin and Thioridazine treatment, respectively (**C**). Nuclei were counterstained with Hoechst (blue). Scale bars, 20 µm. **B:** Immunofluorescence quantification of Prox1 and Ap2α for all conditions indicated a decrease in the percentage of horizontal and amacrine cells after Digoxin and Thioridazine exposure. Data are shown as mean ± SEM, of 5-8 images from different organoids were quantified per condition. Differences were considered statistically significant at *$p < 0.05$, **$p < 0.01$ and ****$p < 0.0001$. Abbreviations: Hoe, Hoechst. Please note IF analysis for Ethanol group was not possible due to sections being fragile.

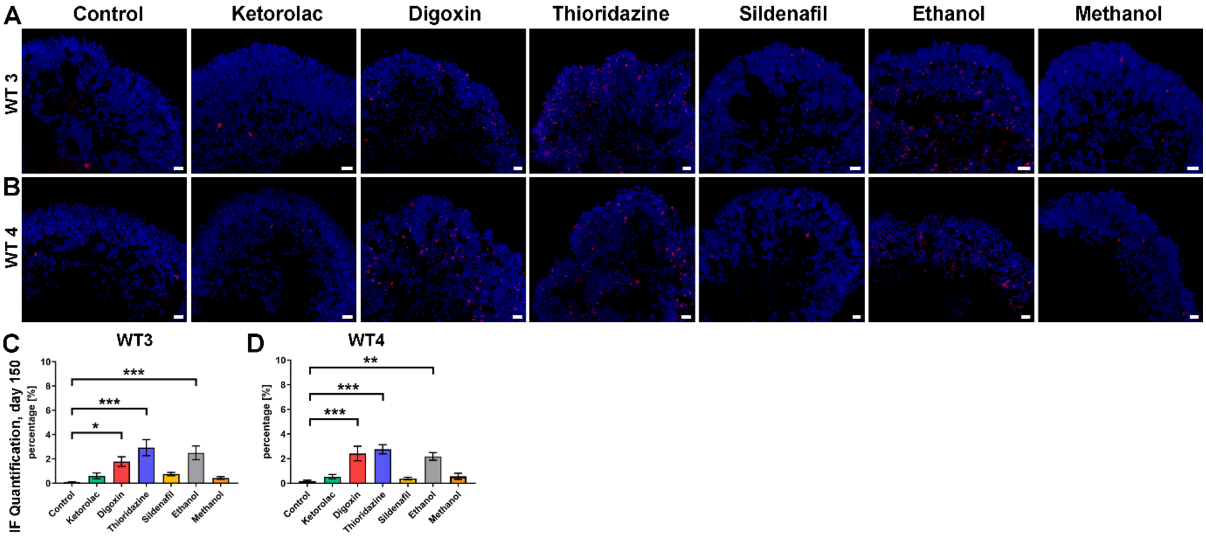

Fig. S6. Increase of cell death after drug exposure in retinal organoids at day 150 of differentiation. **A, B:** Expression of Casp-3 (red) in WT3 derived retinal organoids (**A**) and WT4 derived retinal organoids (**B**), indicated an increase of apoptotic-induced cell death upon Digoxin, Thioridazine and Ethanol treatment. Nuclei were counterstained with Hoechst (blue). Scale bars, 20 µm. **C, D**: IF quantification of Casp-3^{+} cells confirmed significant rise after Digoxin, Thioridazine and Ethanol in retinal organoids derived from WT3 (**C**) and WT4 (**D**). Data are shown as mean ± SEM of 5-8 images from different organoids were quantified per condition. Differences were considered statistically significant at $^{**}p < 0.01$ and $^{***}p < 0.001$. Abbreviations: Hoe, Hoechst.

Table S1: Comparison of statistical significances of WT3 derived retinal organoids using a one-way ANOVA followed by Bonferroni statistical hypothesis for multiple test correction for control versus drugs and Ketorolac versus drugs/compounds (drugs/compounds: Digoxin, Thioridazine, Sildenafil, Ethanol, Methanol; control = PBS treated organoids, Ketorolac = drug control; Abbreviations: Recov, Recoverin; Rho, Rhodopsin).

	Digoxin		Thioridazine		Sildenafil		Ethanol		Methanol	
**Marker**	**Control**	**Ketorolac**	**Control**	**Ketorolac**	**Control**	**Ketorolac**	**Control**	**Ketorolac**	**Control**	**Ketorolac**
**Recov**	**	**	***	***	ns	ns	ns	ns	ns	ns
**Crx**	**	**	*	*	ns	ns	ns	ns	ns	ns
**Rho**	ns	ns	ns	ns	ns	ns	ns	ns	ns	ns
**OPN1 MW/LW**	**	**	ns	ns	*	*	*	*	*	*
**OPN1 SW**	ns	ns	ns	ns	*	*	ns	ns	ns	ns
**G0α**	ns	ns	ns	ns	ns	ns	ns	ns	ns	ns
**Prox1**	***	***	**	***	ns	ns	ns	ns	ns	ns
**Ap2α**	**	***	ns	*	ns	ns	ns	ns	ns	ns
**SNCG**	**	*	ns	ns	ns	ns	ns	ns	ns	ns
**HuC/D**	***	*	*	ns	ns	ns	ns	ns	ns	ns
**Casp-3**	*	*	***	***	ns	ns	***	**	ns	ns
